# ON/OFF and Beyond - A Boolean Model of Apoptosis

**DOI:** 10.1371/journal.pcbi.1000595

**Published:** 2009-12-11

**Authors:** Rebekka Schlatter, Kathrin Schmich, Ima Avalos Vizcarra, Peter Scheurich, Thomas Sauter, Christoph Borner, Michael Ederer, Irmgard Merfort, Oliver Sawodny

**Affiliations:** 1 Institute for System Dynamics, University of Stuttgart, Stuttgart, Germany; 2 Department of Pharmaceutical Biology and Biotechnology, Albert Ludwigs University Freiburg, Freiburg, Germany; 3 Institute of Molecular Systems Biology, ETH Zurich, Zurich, Switzerland; 4 Institute of Cell Biology and Immunology, University of Stuttgart, Stuttgart, Germany; 5 Life Sciences Research Unit, University of Luxembourg, Luxembourg, Luxembourg; 6 Institute of Molecular Medicine and Cell Research, Centre of Biochemistry and Molecular Research (ZBMZ), Albert Ludwigs University Freiburg, Freiburg, Germany; 7 Max-Planck Institute for Dynamics of Complex Technical Systems, Magdeburg, Germany; Université de la Méditerranée & INSERM U928 - TAGC, France

## Abstract

Apoptosis is regulated by several signaling pathways which are extensively linked by crosstalks. Boolean or logical modeling has become a promising approach to capture the qualitative behavior of such complex networks. Here we built a large-scale literature-based Boolean model of the central intrinsic and extrinsic apoptosis pathways as well as pathways connected with them. The model responds to several external stimuli such as Fas ligand, TNF-α, UV-B irradiation, interleukin-1β and insulin. Timescales and multi-value node logic were used and turned out to be indispensable to reproduce the behavior of the apoptotic network. The coherence of the model was experimentally validated. Thereby an UV-B dose-effect is shown for the first time in mouse hepatocytes. Analysis of the model revealed a tight regulation emerging from high connectivity and spanning crosstalks and a particular importance of feedback loops. An unexpected feedback from Smac release to RIP could further increase complex II formation. The introduced Boolean model provides a comprehensive and coherent description of the apoptosis network behavior. It gives new insights into the complex interplay of pro- and antiapoptotic factors and can be easily expanded to other signaling pathways.

## Author Summary

Apoptosis is one of the most investigated topics in the life sciences, especially as this kind of programmed cell death has been linked to several diseases. The strong desire to understand the function and regulation of apoptosis is unfortunately confronted with its complexity and its high degree of cross linking within the cell. Therefore we apply the so-called logical or Boolean mathematical modeling approach to comprehensively describe the numerous interactions in the apoptotic network. Classical Boolean modeling assumes that a certain cellular signal is either present (on) or absent (off). We use extensions of classical Boolean models, namely timescale constants and multi-value nodes, which allow the model to emulate typical apoptotic features. The mathematical model describes for the first time the numerous relevant interactions and signals that control apoptosis in a single and coherent framework. The logical model of apoptosis provides valuable information about the topology of the network including feedback loops and crosstalk effects. Proper investigation of the mutual interactions between species points towards hubs in the network with outstanding relevance. These species are of special interest concerning experimental intervention as well as drug target search. The model we present here is easy to use and freely available.

## Introduction

Apoptosis is the prototype of programmed cell death and an essential process in multicellular organisms. It is necessary during embryogenesis, tissue growth, differentiation and homeostasis as a protective mechanism to remove superfluous or malfunctioning cells from the organism [1]–[5]. Errors in cell death regulation can result in diseases like Alzheimer and Parkinson when uncontrolled apoptosis occurs or cancer if apoptosis is repressed [6],[7]. Apoptosis can be induced by several signal transduction pathways that are tightly regulated and linked to other cellular events such as inflammatory responses and proliferation. The understanding of these signaling pathways is thought to provide novel solutions for the treatment of many diseases. However, a large number of participating components, their complex dependencies and multiple biological stimuli make the analysis of small network parts difficult and often less expressive. Therefore some mathematical models have already been presented covering broader structures.

For example Huber *et al.* presented the web service APOPTO-CELL based on 52 ordinary differential equations [ODEs] to calculate the susceptibility of cells to undergo apoptosis in response to an activation of the mitochondrial apoptotic pathway [8]. The power of ODE based modeling concerning dynamic simulation and system analysis is without controversy. However, the use of ODE models for larger networks is limited due to limited biological data. The parameter identification for ODE models is in the very most cases dependent on quantitative measurements which still are a systems biology bottle neck. Another approach is the use of Petri nets [9],[10], however, the required input for parameterization is still relatively high due to the need of defining transition rules.

In this study, we present a Boolean network of apoptosis. Boolean or logical networks are well suited to reproduce the qualitative behavior of extensive networks even with a limited amount of experimental data. Boolean logic is the algebra of two values, e.g. “1 and 0” or “true and false” or “on and off” [11] and was first shown to be applicable to electrical relay circuits [12]. Furthermore, it can also be applied to biological systems, and signal flow networks can be described reasonable by a logical approach [13]. The Boolean formalism is especially useful for qualitative representation of signaling and regulatory networks where activation and inhibition are the essential processes [14]. In a Boolean representation, the biological active state of a species can be translated into the “on” state whereas the inactive state is represented by the “off” state. Enzymes play the role of switching other enzymes and genes “on” and “off”. Applying Boolean algebra to a signaling network results in an interaction network, analogous to electrical circuits, which can be conveniently represented by logical interaction graphs. Boolean operations and graphs are described in detail in the *Materials and methods* section.

There are different interesting approaches concerning the specific calculation and simulation of Boolean networks. Chaves *et al*. presented a hybrid model of the NF-κB module combining Boolean and ODE based modeling [15]. Calzolari *et al*. analyzed an apoptosis gene network with identical topology but different link strengths chosen by random distribution [16]. For the specific cell type of cytotoxic T lymphocytes Zhang *et al*. built a Boolean model relating the input antigen stimulation with the output apoptosis [17]. They use an asynchronous updating strategy and show multiple simulations with different updating orders. Recently, Mai *et al*. presented a Boolean apoptosis model including 40 nodes and connecting two inputs, namely TNF and growth factor, to the output DNA damage [18]. They calculated their network with the impressive number of 10.000 random initial states to simulate towards apoptosis or stable surviving.

We chose a different approach to avoid some known problems concerning logical models. In this study, the logical steady state [LSS] of variables with a unique LSS for a given input setting is determined. For the computation of LSSs the software tool CellNetAnalyzer [CNA] is used. The propagation of signals through the network is thereby calculated by iterative derivation of partial LSSs for smaller subnets based on already identified partial LSS until no further ones can be found [19]. There is no need to simulate the network many times or to perform statistical analyses. CNA has previously been used to describe and analyze large-scale Boolean models of biological networks. This tool is also useful to predict and verify experimental data, examine the structure and the hierarchy of the system as well as the relevance of its components [19]–[21]. Not least, manual analysis and the identification of network wide dependencies become error-prone for large logical networks. Therefore, construction and analysis of the logical interaction hypergraph model is achieved more reliable in this study using CNA. Special features of the CNA are described in the *Materials and methods* section since they are used to reveal essential properties of the network structure and thereby deduce biological conclusions on the complex signaling network of apoptosis.

**Figure 1:**
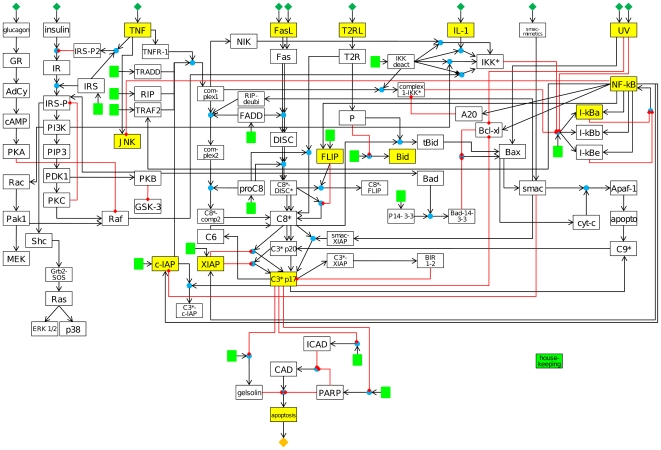
Boolean apoptosis model. The network map as it is also used for CNA is shown. The influence of the housekeeping node is depicted in green color. In addition stimuli and nodes which have been experimentally validated to prove the coherency of the model are indicated by yellow filled background (compare Table 2). Logical AND connections are represented by blue spheres. Activating arcs are represented by black arrows and inhibiting arcs by red lines with a bar.

The large-scale Boolean network constructed in this study is based on extensive literature research. It simulates apoptotic signal transduction pathways in response to various input stimuli and allows a comprehensive evaluation and analysis of the different pathways (Figure 1). We considered the intrinsic and extrinsic apoptotic pathways and their crosstalks as well as the survival and metabolic insulin pathways. We show that the extension and refinement of the logical formalism with multi-value logic and so called timescale constants allows the capturing of dynamical features such as threshold behavior, feedback loops and reaction delays and thereby a correct description of the global signaling behavior. The states of several network nodes are experimentally validated for different inputs in order to prove the coherence of the model. In this context a dose dependent effect of UV irradiation concerning apoptosis induction is demonstrated on mouse hepatocytes. Finally, the model is analyzed with regard to its internal connectivity and crosstalks with a special attention on significant feedback loops and gene regulatory effects.

## Results/Discussion

### General model properties

The model is a logical interaction hypergraph, which is a connection of logic gates, and comprises 86 nodes and 125 interactions (Figure 1). Abbreviations and descriptions of the network nodes are given in Text S1. Text S1 also lists all equations of the model including the respective timescale constants, literature references and organisms from which the information was derived. Due to the number of included interactions in the model we refer to the given literature references for detailed information about the biological processes.

There are eight input nodes, namely glucagon, insulin, TNF-α [TNF], Fas ligand [FasL], interleukin-1β [IL-1], UV-B irradiation [UV] and two special nodes for applying Smac mimetics and for simulating type II apoptotic signaling. Smac mimetics are promising reagents that sensitize cells for apoptosis via the neutralization of inhibitor of apoptosis proteins (IAPs such as XIAP, cIAP1, cIAP2, etc.) [22],[23]. They are considered as a separate node. The input node ‘Type 2 receptor ligand’ [T2RL] allows simulating apoptosis via the mitochondrial type II pathway in contrast to the type I pathway which proceeds via a direct activation of the caspase cascade [24]. The T2RL node is experimentally represented in this study by human Jurkat T cells treated with Fas ligand. Recently, the type I and type II pathways were shown to operate in the same cell type but under different culturing conditions suggesting that cells are able to switch between both ways depending on external stimuli [25]. However, the molecular mechanism of the switch itself has not yet been uncovered. Therefore, an additional node P representing some unknown protein or mechanism is introduced here to model the switch behavior. Another specialty is the ‘housekeeping’ node, which shall reproduce constitutively expressed genes (Figure 1, in green). The output node of the model is apoptosis.

### Timescales facilitate integrated modeling and distinctive analysis

It was shown that dynamic processes can also be captured in logical networks by introducing time delays to the logical functions [13]. An equivalent function is provided in CNA where processes can be assigned to different timescales. These timescales are constants that specify in which state a certain node can become active. Simulating a network at timescale τ = x means that all interactions with a timescale constant τ≤x are considered, but interactions with timescale constant τ>x are omitted. The apoptosis model contains six timescales {τ} = {0, 2, 3, 4, 5, 10} which are not numbered consecutively such that additional timescales can be easily inserted.

Rapid, easily reversible signaling effects like phosphorylation that are based on fast protein interactions can thus be separated from long-term effects like gene expression and protein synthesis. However, we use the so called timescale function not only for an approximate discretization of signaling events to time segments but also to separate functional groups of interactions such as feedback loops. As we calculate the logical steady state, no transition rules for any updating strategy have to be assumed which would be afflicted with high uncertainty. There are no disadvantages connected with extensive defining of timescales concerning the simulation of the network. However, every timescale can be used to generate a snapshot of the network and accomplish its separate analysis. So for example, the topology of the network including only early signaling events or the specific influence of feedback loops can be analyzed by assigning separate timescales to them. Overall the introduction of timescales to the logical formalism allows to describe different signaling effects and gene regulatory mechanisms in one unifying model but to analyze them separately.

All interactions of the apoptosis model with their respective timescales are listed in Text S1. The first timescale τ = 0 is reserved for the housekeeping interactions that activate nodes which are constantly active and represent constitutively expressed genes. Timescale τ = 0 contains 7 interactions and symbolizes the state of the cell before stimulation. However, note that interactions of the housekeeping node with other nodes activated later are set to the later timescale. Also the input and output arcs are assigned to τ = 0 (11 interactions including multilevel inputs). On the second timescale τ = 2 only early TNF signaling events take place which include TNF signal transduction towards the formation of complex I (5 interactions). The internalization of complex I was described to be slow in comparison to other signaling processes. An additional timescale τ = 3 is assigned to further interactions of the TNF pathway that are required for complex II formation (5 interactions). 73 interactions referring to signaling transduction events except the early events of the TNF pathway take place at τ = 4. An additional timescale τ = 5 is introduced to model feedback loops (9 interactions). Assigning a separate timescale to feedback loops allows their separate analysis which is very reasonable considering their impact on the system. The final timescale τ = 10 is reserved for modeling gene expression in response to signaling events and includes 15 interactions.

As an example, some node values for different timescale scenarios after combined stimulation of the apoptosis model with TNF and smac-mimetics are shown in Table 1. All references underlying the according interactions can be found in Text S1. Note that the node complex2 is activated by the interaction RIP-deubi+FADD+comp1 = comp2. The node FADD is set to level 1 by the housekeeping node on timescale τ = 0. At timescale τ = 2 TNF receptor 1 is activated by the input TNF. The input smac-mimetics activates smac and thereby RIP-deubi at timescale τ = 3. At timescale τ = 4 for example the complex smac-XIAP is build. In this setting there is no influence by feedback loops on timescale τ = 5. At timescale τ = 10 upregulation of TRAF2 via NF-κB leads to complex1 formation and thereby to complex2 formation and finally apoptosis.

**Table 1:** Timescale scenarios after combined TNF and smac-mimetics stimulus.

	τ = 0	τ = 2	τ = 3	τ = 4	τ = 5	τ = 10
FADD	1	1	1	1	1	1
TNFR-1	0	1	1	1	1	1
smac	0	0	1	1	1	1
RIP-deubi	0	0	1	1	1	1
smac-XIAP	0	0	0	1	1	1
complex1	0	0	0	0	0	1
complex2	0	0	0	0	0	1
apoptosis	0	0	0	0	0	1

### Modeling of feedback loops

A feedback loop is a circular path in a signed directed graph. In general, feedback loops provide a challenge for Boolean networks as they can lead to oscillations or multistability. In this case no definite logical steady state can be assigned for the affected nodes and they remain unevaluated in CNA. We excluded 13 interactions because of this implication from the logical steady state analysis which are listed and numbered in Text S1. However, these reactions are included in all other computational analysis and can be included in logical steady state computation again very easily by removing the according check mark in the CNA menu *Network Composer*. The omission of these 13 interactions has the benefit of getting a definite simulation result for every possible input setting. Of course the network is changed thereby but this is not disadvantageous as the impact of these particular interactions on the logical steady state analysis is biologically not significant as discussed in the following paragraph.

Four of these 13 interactions represent positive feedback loops which would even enhance an existing activation status in a dynamical model (no. 7–9, 13); however, the respective node is already in the “on” state in the logical model when the feedback would become active. As the Boolean model is not quantitative the feedback loop would not have an impact on the biological result anyway. Three negative feedbacks excluded from logical steady state computation involve the fine-tuning of C3*p17 and Raf activity in a dynamical model, but they do not affect the activation level in the logical model for the same reason (no. 2, 5, 6). Five negative feedback loops govern NF-κB signaling back to its initial configuration and thereby inactivate NF-κB (no. 1, 3, 10–12) and the negative feedback loop towards IRS-P inhibits the signal as well (no. 4). However, the switching off of the network is generally excluded in this model because we restrict ourselves to the critical events of apoptosis. Consequently the respective validation experiments described below are performed in the corresponding time period of the first response of every node.

### Multi-value logic allows threshold behavior

A promising feature of CNA is the possibility to use multi-value logic, which is equivalent to the discretization of the “on” state and was shown to be applicable to logical models of biological systems [13]. Biochemical decisions are often made in increments caused by thresholds that are essential for setting boundaries between different states in living cells. This is especially true for apoptotic processes [26]–[28]. We show here for a comprehensive network that the use of multi-value logic in the description of biological systems allows us to model several distinct active states. Multi-value nodes thereby don't substitute quantitative modeling, but the different node value levels are defined by qualitative properties. This is a general idea of our modeling approach and we name it the functional definition of node values. Assigning different effects to different active states is equivalent to biological threshold behavior. CNA therefore allows the specification of so called non-monotone arcs. In non-monotone interactions multi-value coefficients are assigned to the participating species. Non-monotone interactions can only be active if the specified species coefficients are matched exactly by the species state. For example, consider the two non-monotone interactions 1 A = 1 B and 2 A = 1 C. In this case 1 A will not activate 1 C und also 2 A will not activate 1 B, so the two distinct levels of A can be employed in different further interactions representing different biological effects.

**Figure 2:**
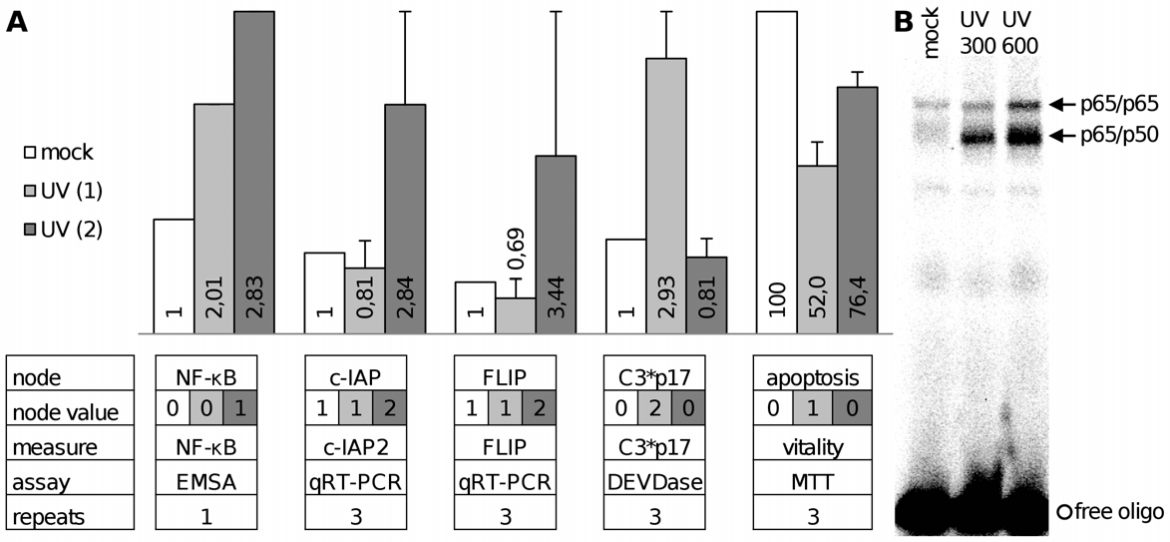
Dose dependent NF-κB activation and apoptosis in response to UV irradiation. Two separate levels for the UV input node are realized in the model. [A] The results for untreated cells and after weak UV (1) and strong UV (2) stimulation for each measure are shown in columns in the upper half including standard deviation. Beneath the diagrams a table lists the according information about the model nodes and the experiments. In particular, the simulation results as predicted by the model for the respective node values are given in the second row. All measures are presented as fold increase and MTT assay results as percentage of vitality referred to untreated control, detailed information about each experimental assay can be found in *Materials and Methods*. [B] The corresponding EMSA as evaluated in Fig. 2A is shown and the NF-κB bands are assigned with arrows.

By default all nodes have been considered as single-value nodes which only occur with the values 0 or 1. Notice that the use of multi-value nodes increases the complexity of the interrelations in the network considerably. However, several biological settings could not be realized with single-value nodes and on that condition the domain of some nodes has been expanded. There are 14 non-monotone interactions in the apoptosis network as listed in Text S1. Non-monotone interactions are involved in the modeling of the FasL pathway, which was reported to show threshold behavior [29] and the modeling of NF-κB mediated upregulation of anti-apoptotic proteins FLIP, XIAP and c-IAPs [30],[31]. The respective multi-value nodes are FasL, Fas, DISC*, FLIP, C8*, C8*-DISC, C3*p20, C3*p17, XIAP and c-IAP that occur with the coefficients {0, 1, 2}. Additionally, a multi-value node for UV irradiation was added based on own experimental results (see Figure 2).

Overall the steady states of the model reflect the following behaviors, which would not be possible without using multi-value nodes: (i) Apoptosis is not reached in the model by FasL in activity state 1 [FasL (1)] but by FasL (2) reproducing the threshold behavior of Fas signaling [26]. However, FasL (1) activates several nodes in the network, and their influence and crosstalk with other signaling pathways can be analyzed. (ii) The nodes of anti-apoptotic proteins FLIP, XIAP and c-IAPs can be set to zero representing a knockout scenario but they also have graded effects in their “on” state. For example, caspase-3 p20 (2) can be further processed to the highly active caspase-3 p17 form which ensues in apoptosis if XIAP is low abundant as it is represented by XIAP (1). However, if XIAP is upregulated to value “2” it prevents processing and activation of caspase-3 p17. (iii) UV (1) leads to apoptosis whereas UV (2) does not lead to apoptosis (see Figure 2).

### Experimental validation of the model

After we built the mathematical model we performed extensive experimental validation. The logical apoptosis model is based on a vast number of different studies, which were performed in different organisms and were in part highly focusing on important details. Here, we show that the behavior that emerges from these particular interactions in the model is coherent with experimental data on the behavior of the whole network. Table 2 shows the model prediction for different proteins and stimuli which are critical for apoptosis represented by the resulting logical steady state values of the model for the final timescale τ** = **10. The model values of the input nodes are given in parentheses in Table 2 and mock is represented by the logical steady state of the model without activation of any input node.

**Table 2:** Central results of the logical apoptosis model have been experimentally validated.

		NF-κB	IκB-α	c-IAP	XIAP	C3*p17	FLIP	Bid	JNK	apoptosis
celltype	stimulation	Emsa	Western	qRT-PCR	Western	DEVD	qRT-PCR	Western	Western	MTT
jurkats	mock	0	1	1	1	0	1	1	0	0
jurkats	T2RL (1)	0	1	1	1	2	1	0	0	1
hepatocytes	mock	0	1	1	1	0	1	1	0	0
hepatocytes	FasL (2)	0	1	1	1	2	1	1	0	1
hepatocytes	TNF (1)	1	0	2	2	0	2	1	1	0
hepatocytes	IL-1 (1)	1	0	2	2	0	2	1	0	0
hepatocytes	UV (1)	0	1	1	1	2	1	1	0	1
hepatocytes	UV (2)	1	0	2	2	0	2	1	0	0

In the experiments, two different cell types were used to account for the distinct signaling mechanisms in cells using the type I (mouse hepatocytes treated with FasL) and the type II (human Jurkat T cells treated with FasL representing the T2RL node) apoptotic pathways. The measured parameters/nodes of the model are: NF-κB-DNA binding and IκB-α degradation for NF-κB-signaling, activated caspase-3 [C3*p17] and the mRNA levels of inhibitory proteins c-IAP, XIAP and FLIP for the caspase cascade, Bid as member of the Bcl-2 family, the activation state of c-Jun N-terminal kinase [JNK] and finally apoptosis as an output signal. Note that the different forms of c-IAPs and FLIP are merged to one node in the model, and measured mRNA levels are c-IAP2 and all 3 isoforms of FLIP. The respective stimuli and nodes are also indicated in Figure 1. Details on the experimental procedures can be found in the section *Materials and methods*.

**Figure 3:**
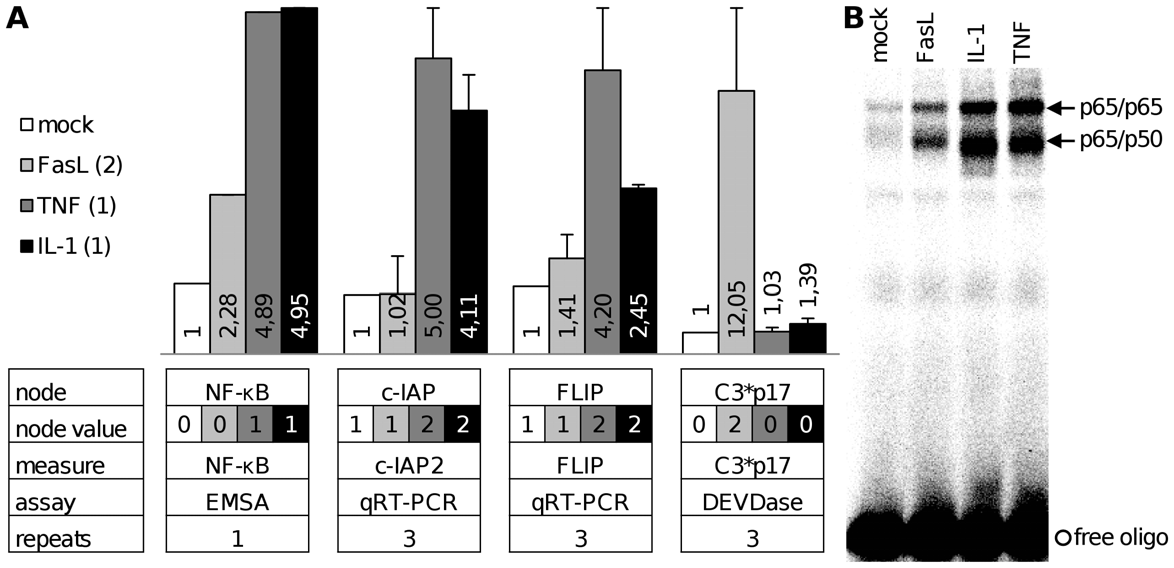
Experimental model validation for stimulation with Fas ligand, TNF-α and interleukin-1β. Primary mouse hepatocytes were treated with FasL, TNF-α or IL-1β. [A] The results are shown in columns in the upper half including standard deviation. Beneath the diagrams a table lists the according information about the model nodes and the experiments. In particular, the simulation results as predicted by the model for the respective node values are given in the second row. All measures are presented as fold increase and MTT assay results as percentage of vitality referred to untreated control, detailed information about each experimental assay can be found in *Materials and Methods*. [B] The according EMSA as evaluated in Fig. 3A is shown and the NF-κB bands are assigned with arrows.

All model predictions listed in Table 2 were successfully approved on the first try without changing the model, apart from the effect of UV irradiation on the network. We found an unexpected UV dose effect in primary mouse hepatocytes which was included in the model and will be discussed in the next section. First, the system response to FasL, TNF-α and IL-1β will be presented. All measured entities could be experimentally shown to be active or existing, respectively inactive or non-existing as predicted by the model in response to these stimuli. Selected results of FasL, TNF-α and IL-1β stimulation in mouse hepatocytes are shown in Figure 3. Stimulation with FasL leads to only weak NF-κB activation and hence no significant c-IAP2 and FLIP upregulation. As there is no signaling effect on the subsequent nodes the model shows NF-κB (0) in this setting according to the functional definition of its node value which is depending on the node's effect on the network. Caspase-3 p17 is highly active. In contrast, NF-κB is clearly activated after stimulation with TNF-α or IL-1β. Accordingly, c-IAP2 and FLIP are upregulated and, as predicted, caspase-3 p17 is not activated.

All validation experiments for Table 2 which are not shown in Figure 3 can be found in Protocol S1. In addition we tested apoptosis induction in Jurkat T cells after stimulation with TNF-α and IL-1β. As expected and predicted by the model these stimuli do not have cytotoxic effects on the cells and the according experiments are documented in Protocol S1. It is impossible to test every signaling scenario of the presented apoptosis model due to technical limitations and the mere number of nodes. However, the accuracy of the performed validation experiments indicates fundamental correctness and significance of the model.

### UV irradiation triggers dose dependent NF-κB activation and apoptosis

During experimental validation of the model, we found dose dependent NF-κB activation and apoptosis after UV irradiation in primary mouse hepatocytes. Based on the results shown in Figure 2, two distinct levels for the UV input node were implemented. The updated model version properly reflects the network behavior in response to UV irradiation and is presented here.

UV (1) represents the stimulation of mouse hepatocytes with 300 J/m^2^ UV irradiation and UV (2) with 600 J/m^2^. Weak UV irradiation leads to weak NF-κB activation and no c-IAP2 and FLIP mRNA upregulation. As there is no signaling effect on the subsequent nodes the model shows NF-κB (0) in this setting. As a consequence, mouse hepatocytes show significantly increased caspase-3 p17 activity and consequently cytotoxicity due to apoptosis can be observed as expected after UV irradiation. In contrast, the higher dose of UV irradiation leads to strong NF-κB activation and subsequently c-IAP2 and FLIP mRNA is upregulated. This correlates with previous findings showing a marked NF-κB induction after strong translational inhibition and relative resistance to lower doses [32]. The proteins c-IAP2 and FLIP function as anti-apoptotic inhibitors and prevent caspase-3 p17 activity in this setting. Accordingly, cells show less cytotoxicity after strong UV irradiation and the amount of cell death observed in the MTT viability assay is probably caused to a high extent by necrosis when comparing with caspase-3 p17 activity. In addition, we also treated Jurkat T cells with UV irradiation. We did observe apoptosis neither after 300 J/m^2^ nor after 600 J/m^2^ and expect the critical apoptotic UV irradiation dose for Jurkats at higher levels. All validation experiments concerning UV irradiation which are not shown in Figure 2 can be found in Protocol S1.

### 95 feedback loops including an unexpected one

A feedback loop is a circular path in a signed directed graph, and the overall sign of F is determined by the parity of the number of inhibiting and activating arcs [33]. The sign of a feedback loop has great impact on the dynamics of a system. On the one hand, positive feedback loops allow for multistationarity which is required for epigenetic differentiation in biological systems [34]–[36]. On the other hand, negative feedback loops generate periodicity and are essential for maintaining homeostasis [35],[36].

**Figure 4:**
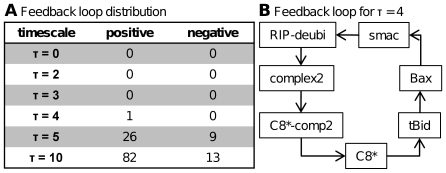
Feedback loops in the apoptosis network for different timescale constants τ. [A] The distribution of positive and negative feedback loops for all timescale constants τ is listed. [B] An unexpected feedback loop arises in the model for τ** = **4. Complex II activates caspase-8 which leads to the release of Smac in response to Bid cleavage. Smac could promote complex II formation by increasing the amount of available RIP-deubi.

The total number of positive and negative feedback loops for each timescale is shown in Figure 4A. As CNA searches for feedback loops of arbitrary length in the network the algorithm finds in fact more feedback loops as expected from a superficial look on the network map. Considering the interactions for τ = 5 there are already 26 positive and 9 negative feedback loops. For τ = 10 these numbers increase up to 82 positive and 13 negative feedback loops. This proportion reflects the typical features of apoptosis networks where positive signal amplification and multistationarity are characteristic. In contrast, antiapoptotic mechanisms are rather realized by inhibitory proteins such as XIAP than by negative feedback loops.

Interestingly, as shown in Figure 4B, there is an unexpected feedback already for τ = 4 in the network which was not modeled explicitly. The formation of complex II induces activation of caspase-8 which leads to the release of Smac in response to Bid cleavage finally resulting in mitochondrial pathway activation in type II cells. According to our model, Smac could further increase complex II formation by increasing the amount of available RIP-deubi. The biological relevance of this feedback is speculative. However, the topological possibility of a feedback loop in apoptosis signaling upstream of the caspase cascade is fascinating and potentially important.

The relevance of feedback loops [37]–[39] and associated affects such as bistability [27],[40] and oscillations [41],[42] are a largely discussed topic. The so far analyzed and well known feedback loops are usually consisting of very few molecules [43],[44]. The analysis of the apoptosis model shows a high number of feedback mechanisms consisting of many interactions building long loops. As the Boolean model is not dynamic it cannot tell whether these structures are biological relevant or take place on an insignificant timescale. However, their further analysis might be promising.

### Feedback loops are essential for signaling towards apoptosis

In the following section, we discuss the influence of feedback loops and gene regulatory effects on the signaling behavior of the model for τ = 5 and τ = 10. The relative participation of network components in all feedback loops on the respective timescale is shown in Text S1. The general tendency of signaling is still maintained for τ = 5 as the apoptosis supporting input nodes mainly participate in positive signaling pathways and vice versa. However, the combination of negative and positive pathways allows for a more differentiated response to input signals. The components of the caspase module are involved in most of the feedback loops for τ = 5, and their relative participation reaches up to 89% for C3*p17 (Text S1). This high involvement originates from the high connectivity of these nodes with other pathways and is indicative of the important role of caspases, especially caspase-3, in apoptosis regulation. For τ = 10 we noticed an increased involvement of NF-κB and components of the mitochondrial module in feedback regulation. In particular, Bax participates at 76% (Text S1). In summary, only a small group of species is involved in most of the feedback loops, but as such this group plays a prominent role in the regulation and determination of the network response to input signals. This small group consists mainly of caspases, mitochondrial proteins and NF-κB signaling components which are important for the robustness of the entire system and indicate their importance in apoptosis execution and control.

The regulatory importance of feedback loops is also reflected by the species dependencies for different timescales. The respective dependency matrices are due to their size shown in Figures S1, S2, S3. Until τ = 4 almost only total activation and inhibition processes occur in the network which represents the linear and parallel behavior of the signaling processes (Figure S1). A comparison with the species dependencies for τ = 5 shows a substantially changed network topology and reveals all species that are influenced by negative feedback loops in their respective pathways but also pathways to which they are connected (Figure S2). The dependency matrix for τ = 10 finally completes the overall picture of complex and ambiguous relationships in the network showing almost no total activation and inhibition processes anymore but an increased number of ambivalent effects (Figure S3).

The total number of calculated signaling pathways from each start node to the apoptosis node is shown in Table 3 for each timescale. No continuous signaling pathways to the apoptosis node exist for τ≤3 because the caspase activation module is only active for τ≥4 as described before. For τ = 4 all input nodes with apoptosis supporting effects exclusively participate in positive signaling pathways to the apoptosis output node. In accordance, all input nodes with apoptosis inhibiting effects do not show any or only negative pathways. This topology describes a non-regulated cell which would show a linear signaling behavior without the ability to integrate received information and adapt to situations. Additionally, the constraint signaling behavior would render the cell error-prone for the failure of individual molecular species. For τ = 5 feedback loops extend the network topology. Although only nine interactions are added at this timescale their impact is significant and most input nodes already have ambivalent potential to influence the apoptosis node depending on further circumstances. The number of signaling paths from the input nodes to apoptosis finally dramatically increases for τ = 10 by adding gene regulatory effects by the NF-κB node. Concerning the final decision between cell survival and apoptosis the overall network presents itself as highly crosslinked and regulated in a complex manner.

**Table 3:** Signaling pathways from every input node to the apoptosis node for all timescales τ.

input node	τ = 0	τ = 2	τ = 3	τ = 4		τ = 5		τ = 10	
				positive	negative	positive	negative	positive	negative
FasL	0	0	0	704	0	704	0	1216	0
glucagon	0	0	0	0	0	0	0	192	224
IL-1	0	0	0	0	0	0	0	224	192
insulin	0	0	0	0	44	68	44	696	748
smac-mimetics	0	0	0	44	0	44	32	236	32
T2RL	0	0	0	88	0	88	24	248	24
TNF	0	0	0	88	0	88	24	792	1080
UV	0	0	0	88	0	88	48	568	240

### High connectivity and crosstalks are significant for apoptosis signaling

High connectivity increases the number of possible pathways between two nodes and the reliability and flexibility of the network to respond to its environment. CNA considers strongly connected components as maximal subgraphs of the interaction graph in which paths between all pairs of nodes exist. The apoptosis model contains two groups of strongly connected components. One comprises the nodes PKC, PKB, PDK1, PIP3, PI3K and IRS-P. These nodes are part of the insulin signaling pathway and connected to a feedback loop by PKB. The second group contains 30 nodes, which belong to complex formation in the upper apoptosis signaling (complex1, complex2, TRAF2, RIP-deubi, comp1-IKK*, NIK, C8*-comp2, FLIP), caspase cascade (C6, C3*p20, C3*p17, C3*-XIAP, XIAP, c-IAP, C8*, C9*, BIR1-2), mitochondrial release (tBid, Bax, Bcl-xl, apopto, Apaf-1, smac-XIAP, smac, cyt-c) and NF-κB signaling (NF-κB, IκB-α, IκB-ε, A20, IKK*). The high connectivity between these nodes is only partially due to the cascading topology of enzyme activation. Furthermore, the involved proteins such as the inhibitor XIAP, several feedback loops and especially the inclusion of NF-κB signaling in this strongly connected subgraph reflect the highly controlled and robust structure of death signaling.

As a transcription factor, NF-κB has central role for the network. The anti-apoptotic impact of NF-κB is ensured via the upregulation of survival factors. However, analysis with CNA reveals an even broader influence of the NF-κB node resulting from its high connectivity. There are 34 inhibitors, 27 activators and 8 ambivalent factors affecting NF-κB. In turn, NF-κB is an ambivalent factor for 30 species, an activator for 8 and an inhibitor for 1.

In addition to these highly connected subgraphs crosstalks between individual signaling modules determine the behavior of the network. Amongst others, the model includes the following crosstalks with insulin signaling (documented with the according interactions in Text S1): (i) TNF-α stimulates IRS phosphorylation and thereby inhibits insulin signaling. (ii) In response to insulin PKB is activated and phosphorylates Bad. Phosphorylated Bad is sequestered by 14-3-3 proteins and therefore cannot activate pro-apoptotic Bax. (iii) PI3K is involved in insulin signaling and also contributes to NF-κB activation via IKK. (iv) Raf can be activated via insulin signaling and inhibited by glucagon signaling and active Raf also triggers IKK-dependent NF-κB activation. Also there were two crosstalks explicitly presumed in the modeling process. Smac mimetics were shown to have an apoptosis promoting effect after stimulation with TNF-α [23] and also lead to autocrine TNF-α secretion [45],[46]. The network reflects this crosstalk as Smac mimetics don't induce apoptosis but promote complex II building via RIP and lower the threshold for C3*p17 activation via sequestering XIAP. Accordingly, while TNF stimulation of the model does not lead to apoptosis as observed in hepatocytes and Jurkat T cells, the combination of TNF and Smac mimetics does. Another crosstalk is based on the antiapoptotic influence of IL-1β via NF-κB [47]. Although FasL (2) alone leads to apoptosis it does not in combination with IL-1β (1) in the model.

The explicitly and implicitly modeled crosstalk connections in the network also lead to further effects in the model. The resulting value for the apoptosis node is systematically simulated for all double stimulation scenarios and listed in Table 4. The diagonal shows the resulting apoptosis value for the according single stimulations. One would assume the outcome for two combined stimuli to follow the rules 0+0 = 0, 1+1 = 1 and 0+1 = 1. However, there are some aberrations which are highlighted bold in the Table and discussed in the following text. Smac-mimetics lead to apoptosis in combination with FasL (1) by the same mechanism as discussed above. There are also two other combinations aside from IL-1β which prevent apoptosis after FasL (2) stimulation in the model. Namely Insulin and TNF have an antiapoptotic effect based on NF-κB activation via Raf and complex-1 respectively. There are also some interesting crosstalks concerning UV stimulation. The antiapoptotic effects of insulin and IL-1β also prevent apoptosis in combination with UV (1). However, in combination with TNF apoptosis is still enforced by UV (1) as smac is released by UV irradiation and counteracts XIAP upregulation. The input combinations of UV (2) with TNF and FasL (1) also lead to apoptosis as the latter activate caspase-8 (1). In contrast, the combination of FasL (2) and UV (2) does not cause apoptosis in the model as the NF-κB activation by UV (2) is dominant in this setting.

**Table 4:** Apoptosis node value for all double stimulation scenarios of the model.

	Glucagon	Insulin	TNF	FasL (1)	FasL (2)	T2RL	IL-1	smac-mimetics	UV (1)	UV (2)
Glucagon	0	0	0	0	1	1	0	0	1	0
Insulin		0	0	0	**0**	1	0	0	**0**	0
TNF			0	0	**0**	1	0	**1**	1	**1**
FasL (1)				0	–	1	0	**1**	1	**1**
FasL (2)					1	1	**0**	1	1	**0**
T2RL						1	1	1	1	1
IL-1							0	0	**0**	0
smac-mimetics								0	1	0
UV (1)									1	–
UV (2)										0

In the future we will especially focus on the investigation and expansion of the model regarding further crosstalk effects between distinct pathways as well as on their experimental validation. Unfortunately, this is not trivial as the Boolean model does not give advice how to combine stimuli experimentally concerning timing and dosage. However, the connectivity of subnetworks and single components via crosstalks is helpful information to include all essential interactions when focusing on a smaller subsystem or specific question. We propose to check the Boolean model for important interaction players when modeling a particular signaling pathway or designing biological experiments to elucidate functional relationships.

### The logical apoptosis model may support your research

The Boolean approach we use here for modeling apoptosis obviously has a systematic drawback resulting from the reduction on qualitative network behavior. The reaction rates of biological processes and the quantitative amount of molecules cannot be assigned straight forward to model values. Instead careful conversion has to be done for particular cases and biological knowledge of the modeler is of special importance. In return the presented logical model is easy to use and very flexible.

Protocol S2 comprises detailed instructions how to start up the apoptosis model (Protocol S3) without any previous knowledge. One can use the apoptosis model for comparison with own results as well as for further analyses. It can be modified and expanded to other cell types, additional pathways or crosstalks. In particular, any kind of knock-out or knock-in scenario can be simulated with the model by setting certain nodes or interactions to the desired value. Subsequently, resulting variations in signaling behavior and the changed network topology can be analyzed. On the other hand CNA can search for minimal intervention sets. Thereby the algorithm computes all possibilities to reach a user-defined network state under user-defined constraints as fixed states or maximum number of interventions. Finally, uncovering sensitive points in the network and failure modes of the system concerning specific questions will provide suggestions for biological experimental design as well as predictions how the system reacts in response to selected challenges.

Taken together, the logical model presented here can easily be applied to a broad spectrum of scientific questions concerning apoptosis signaling pathways and their complex crosstalk to other pathways and serve as a helpful and valuable tool in a variety of research aims.

## Materials and Methods

### Interaction graphs

Each species of a network is considered to be a node and two nodes are connected by an edge, also called arc, indicating a direct dependency between them. Nodes and edges form a graph. Directed graphs are a subclass of graphs in which the orientation of the edge determines the direction of the signal flow [48]. At the boundaries of an interaction graph sources and sinks can be found. Sources represent inputs and are not influenced by other nodes. Sinks represent outputs and do not influence further nodes.

Adding a sign to the edge specifies whether the influence of a node is activating (positive) or inhibiting (negative). In signed directed graphs linear connections between two nodes that are not directly connected to each other describe paths which have an overall sign. The sign of the sequence of arcs is negative if the number of arcs with negative sign is odd and positive if the number of arcs with negative signs is even or zero.

### Feedback loops

Feedback loops in the biological sense are regulatory functions that integrate the state of a downstream system variable with a state prior in the path and return an answer which then leads to further enhancement or abortion of the signal. In a graph theoretical sense a feedback loop would involve only one node influencing itself. In this work the term feedback loop is used in the biological sense involving one or more nodes. A feedback loop ends at the same node where it started and no other node is visited twice. The overall sign of a feedback loop is determined by the parity of the number of inhibiting and activating arcs [33]. The sign of a feedback loop has great impact on the dynamics of a system [34]–[36].

### Boolean logic operations

Logical counterparts to numerical operations are conjunction, disjunction and complement. This will be explained for the example of two variables A and B. A numerical multiplication is analogous to a logical conjunction expressed by (A Λ B) or (A AND B). A conjunction of two statements is true when both statements are true. A numerical addition of A and B is expressed in Boolean algebra as a disjunction (A V B) or (A OR B). A disjunction of two statements is true when one of the statements is true (inclusive disjunction). In the presented logical apoptosis model disjunctions are not notated explicitly but are represented by several interactions which can lead to the same result. A numerical negation (–A) is expressed by (A), (NOT A) or (!A) in Boolean algebra. The complement of a statement is true when the statement is false.

### Hypergraphs

Another important property of biological regulatory networks is the participation of two or more species in one interaction whereas in an interaction graph one node influences one other node. A representation of more than one species influencing another can be facilitated by logical AND connections. A graph containing AND connected species is a hypergraph [49]. A hyperarc connects two subsets of nodes. The resulting graph is termed a logical hypergraph [19].

### CNA/ProMoT

The MATLAB based tool CellNetAnalyzer [CNA] [19] allows construction and analysis of metabolic (stoichiometric) as well as signaling and regulatory networks via a graphical user interface. In this study CNA Version 9.2 has been used. The network map can be created with external programs, and we used Microsoft Power Point.

A Boolean network is represented in CNA as a logical interaction hypergraph that can also be transformed into an interaction graph. Thereby hyperarcs are splitted and parallel arcs can arise which may lead to undesired effects. For example, (A+B = X, A+C = X) is converted to (A = X, B = X, A = X, C = X). After transformation of the presented logical apoptosis model four duplicated arcs and ten parallel edges are removed. The interaction graph representation is required for computation of signaling pathways, feedback loops and species dependencies because it unambiguously indicates which nodes are involved in interactions. For logical steady state analysis and minimal intervention sets the logical interaction hypergraph representation is required to capture all constraints and influences included in the model.

### Computation of feedback loops and signaling paths using CNA

The CNA algorithm for computation of feedback loops identifies paths with the same start and end node. Additionally, the direction of the edges is considered so that signal flow occurs only in the specified direction. Another necessary property of the calculated circuits is their non-decomposability into smaller circuits in order to fulfill the notion of elementary modes [50]–[52]. Signaling pathways in CNA are calculated in analogy to feedback loops.

### Computation of network wide dependencies using CNA

To identify the influence a species A exhibits on another species B all signaling paths leading from A to B can be computed. A is not influencing B if such a path does not exist. Otherwise the influence of A on B is characterized as follows: A is a total activator/inhibitor of B if only activating/inhibiting paths are found. A is a non-total activator/inhibitor of B if only activating/inhibiting paths are found but a path contains an intermediate node that is involved in a negative feedback loop. A is an ambivalent factor if activating and inhibiting paths are found.

### Computation of the logical steady states using CNA

For every Boolean network all possible logical steady states [LSSs] can be calculated [53]. In CNA a LSS is computed based on specified initial values and the signal propagation through the network is calculated. There are no interactions with so called incomplete truth tables in the network so that all nodes can be evaluated for every input setting. LSSs can be used to simulate changes in the network structure and analyze the consequences on the signal propagation. The knock-out of a certain gene is represented by deactivation or removal of a species achieved by setting the value of this species to zero. Constitutive expression of a gene can be represented by setting the value of this species to greater zero (on-state).

### Cell culture, isolation and cultivation of primary mouse hepatocytes

Primary hepatocytes were isolated from 8–12 week old B6 (C57Bl/6NNrl) mice as previously described [54]. The use of mice for hepatocyte isolation has been approved by the animal experimental committees and animals were handled and housed according to specific pathogen free (SPF) conditions. Cells were plated on collagen-coated tissue culture dishes in William's medium E (WME, from Biochrom) supplemented with 10% FCS, 100 nM dexamethasone, 2 mM L-glutamine and 1%-penicillin/streptomycin solution (all reagents from Gibco). Cultivation was carried out as described [54], following a three step starvation procedure. To allow hepatocytes to attach, cells were kept in a humidified atmosphere at 37°C and 5% CO_2_ for 4 h. Subsequently, FCS cell culture medium was removed and replaced by serum-free culture medium (WME supplemented with 100 nM dexamethasone, 2 mM L-glutamine and 1%-penicillin/streptomycin solution). Following 4 h incubation in serum-free culture medium hepatocytes were washed three times with starvation medium (WME supplemented with 2 mM L-glutamine and 1%-penicillin/streptomycin solution) and further kept for 16–24 h in the same medium.

Jurkat T cells (suspension) were maintained in RPMI 1640 medium supplemented with 10% FCS and 1%-penicillin/streptomycin.

### Preparation of total and nuclear cell lysates

For preparation of total extracts 2×10^6^ cells were centrifugated (2150 g, 4°C, 3 min), washed with PBS, centrifugated again and 140 µl of lysis buffer (136 mM NaCl, 2 mM EDTA, 20 mM Tris/HCl pH 7.4, 10% glycerol, 4 mM benzamidine, 50 mM β-glycerophosphate, 20 mM Na-diphosphate, 10 mM NaF, 1 mM Na_3_VO_4_) supplemented with protease inhibitors (5 µg/ml aprotinin, 5 µg/ml leupeptin, 0.2 mM pefablock) was added. Cell lysis was performed by shaking for 20 min at 4°C and final centrifugation at 20800 g, 4°C for 10 min.

For preparation of nuclear extracts 1×10^6^ cells were washed with PBS and collected in Eppendorf tubes. After centrifugation (2150 g, 4°C, 3 min), the pellet was resuspended using 400 µl buffer A (10 mM Hepes/KOH pH 7.6, 15 mM KCl, 2 mM MgCl_2_, 0.1 mM EDTA pH 8.0) and incubated on ice for 10 min. Then, the cell suspension was centrifuged (2150 g, 4°C, 3 min) and buffer A was replaced by 200 µl buffer A containing 0.2% NP-40 supplemented with Complete protease inhibitors (Roche Applied Science) and incubated for exactly 5 min on ice to lyse the cytoplasma membrane. After centrifugation (8062 g, 4°C, 2 min), supernatants were stored as cytoplasmic extracts and pellets were resuspended in 50 µl buffer C (25 mM Hepes/KOH pH 7.6, 50 mM KCl, 0.1 mM EDTA pH 8.0, 10% glycerol, Complete protease inhibitors) and kept on ice. After 5 min, 4.5 µl of a 5 M NaCl solution was added and incubated for 30 min with gentle shaking at 4°C. After centrifugation (20800 g, 4°C, 10 min) the supernatant was isolated as nuclear extract.

### DEVDase assay

For measuring the activity of the executioner caspases 3/7 DEVDase assay was performed. Primary mouse hepatocytes and Jurkat T cells (1×10^6^ cells respectively) were incubated with TNF-α (R&D Systems) 25 ng/ml, IL-1β (Jena Bioscience) 50 ng/ml or FasL (N2A FasL as described in [25]) 50 ng/ml for 6 h or exposed to 300 J/m^2^ or 600 J/m^2^ UV irradiation (Stratalinker UV crosslinker from Stratagene). Then the cell suspension was centrifugated, washed with PBS and homogenized in 50 µl of homogenization buffer. Caspase-3 activity assay was performed exactly as described in [55] using the caspase-3 substrate DEVD-AMC (Alexis) at a concentration of 200 nM. Relative fluorescence units (RFU) values were calculated via the ratio of average rate of the fluorescence increase and protein concentration determined by Bradford assay (Biorad). To compare different experiments, RFU sample values were referred to negative control (untreated cells). At least three independent experiments were carried out and means of these experiments including the SD are shown.

### MTT viability assay

After exposition to the different stimuli for 6 h or to UV irradiation of the aforementioned doses, primary hepatocytes and Jurkat T cells were treated with 1 ml of 0.5 mg/ml MTT (Sigma) solution in PBS, and incubated at 37°C for 2 h. After observing a color change to purple the supernatant was removed and the crystals dissolved in DMSO. The samples were transferred into a fresh 96-well plate, and the color reaction measured with an ELISA reader at 595 nm. The sample values were referred to untreated control. Again, means of three independent experiments with SD are shown. Please note that the MTT assay only measures viability and does not differentiate between apoptosis and other forms of cell death.

### Electrophoretic mobility shift assay (EMSA)

Nuclear protein extracts were prepared as described above. Equal amounts of nuclear proteins (4 µg) were added to a reaction mixture containing 20 µg bovine serum albumin, 2 µg poly(dI-dC) (Roche Molecular Biochemicals), 2 µl buffer D+ (20 mM HEPES, pH 7.9, 20% glycerol, 100 mM KCl, 0.5 mM EDTA, 0.25% NP-40, 2 mM DTT, 0.1% PMSF), 4 µl buffer F (20% Ficoll 400, 100 mM HEPES, 300 mM KCl, 10 mM DTT, 0.1% PMSF) and 100,000 cpm (Cerenkov) of a P33-labeled oligonucleotide for NF-κB made up to a final volume of 20 µl with distilled water. For competition experiments (not shown) the reaction mixture contained a 100-fold excess of the respective non-radioactive labeled oligonucleotide. NF-κB oligonucleotide (5′-AGT TGA GGG GAC TTT CCC AGG C-3′, Promega) was labeled using [γ33P]ATP (3000 Ci/mmol, Amersham Biosciences) and a T4 polynucleotide kinase (New England Biolabs). After 25 min of incubation at room temperature the samples were resolved through non-denaturing 6% polyacrylamide gel electrophoresis and then the dried gel was exposed to an Imaging Plate (BAS-MS 2340, Fujifilm) overnight which was finally analyzed using a FLA-3000 (Fujifilm). In the figures the resulting images are shown together with the quantified 33P-stimulated luminescence (PSL) units of each specific shift. Dimer composition was determined by supershift analysis (not shown) using specific antibodies for p65 and p50 NF-κB subunits (from Santa Cruz Biotechnologies).

### Western blotting

To analyze protein levels in total cell lysates, samples containing 50–70 µg protein were separated by SDS-PAGE (12% or 15% gels) and transferred to a 0.45 µm or 0.2 µm pore size PVDF (Roche Applied Science and BioRad, respectively) membrane. Antigen detection was done using antibodies against P-JNK at 1∶1000 (Cell Signaling), IκB-α at 1∶1000 (Cell Signaling), β-actin at 1∶10000 (MP Biomedicals), Bid at 1∶700 (gift from David Huang, WEHI), XIAP at 1∶2000 (StressGen), appropriate horseradish peroxidase-labeled secondary antibodies (Jackson ImmunoResearch Laboratories or Cell Signaling), and the ECL plus chemiluminescence detection reagent (Amersham Biosciences). Chemiluminescent images were quantified using the LumiImager and the LumiAnalyst Software (Roche Applied Science).

### RNA isolation, cDNA synthesis and qRT-PCR

Total RNA was isolated using RNeasy Plus Kit (Qiagen) and extraction was performed according to the manufacturer's directions. The quantity and purity of RNA was determined by measuring the optical density at 260 and 280 nm. Subsequently, 1 µg of total RNA was converted to single strand cDNA using Quantiscript Reverse Transcriptase (Qiagen) resulting in 100 µl diluted cDNA. The analysis of mRNA expression profiles was performed with multiplex quantitative real time PCR. In a 25 µl PCR reaction, 2 µl of cDNA (corresponding to 20 ng of total RNA input) was amplified in an Light Cycler 480 (Roche), using 2-fold QuantiTect Multiplex PCR Master Mix (Qiagen), 50 nM primers and 100 nM probe for the 18S rRNA reference gene (fwd: 5′-CGGCTACCACATCCAAGG-3′, rev: 5′-CGGGTCGGGAGTGGGT, probe: 5′-TTGCGCGCCTGCTGCCT), and 300 nM primers and 100 nM probe for the gene of interest. The following target gene primers and probes were used (all from Sigma): mouse cIAP2 (fwd: 5′-ACATTTTCCCCACTGTCCATTT-3′, rev: 5′-CTATCCAGGGGTCATCTCCA-3′, probe: 5′-ATGCAGACACACTCTGCTCG-3′), human cIAP2 (fwd: 5′-CTGGAAACAAAGCATTGAAGTCTG-3′, rev: 5′-GCCATTAGTAAAGAGGTTCTGAGTC-3′, probe: 5′-CGTCTGTGAGATCCAGGAAACCATGCTTGC-3′), mouse cFLIP (fwd: 5′-TGCCAGAGTGTGGAGAACAG-3′; rev: 5′-TTACCCAGTCGCATGACAAA-3′; probe: 5′-GGGGGAGGTTATCTACCAAGT-3′) and human cFLIP (fwd: 5′-AGACCCTTGTGAGCTTCCCTAG-3′, rev: 5′- GCAGCATCTCCTTCTCATCTGTATC-3′, probe: 5′-AGTGCTTCTTCAACCTGATGGATGACTTCA-3′). The mRNA level for the gene of interest was determined as 2-ΔΔCT and therefore reflects changes relative to unstimulated cells. Cells were treated with TNF-α 25 ng/ml, IL-1β 50 ng/ml or FasL 50 ng/ml for 8, 3 or 6 h respectively. All experiments were performed at least three times and means of three independent experiments with SD are shown.

## Supporting Information

Figure S1 The dependency matrix for τ = 4 displays the influence of each node on each other node in the network. Legend: dark green: A is total activator of B, dark red: A is total inhibitor of B, yellow: A has activating and inhibiting effect on B, black: no influence of A on B, light green: A is non-total activator of B, light red: A is non-total inhibitor of B.
(0.18 MB TIF)

Figure S2 The dependency matrix for τ = 5 displays the influence of each node on each other node in the network. Legend: dark green: A is total activator of B, dark red: A is total inhibitor of B, yellow: A has activating and inhibiting effect on B, black: no influence of A on B, light green: A is non-total activator of B, light red: A is non-total inhibitor of B.
(0.19 MB TIF)

Figure S3 The dependency matrix for τ = 10 displays the influence of each node on each other node in the network. Legend: dark green: A is total activator of B, dark red: A is total inhibitor of B, yellow: A has activating and inhibiting effect on B, black: no influence of A on B, light green: A is non-total activator of B, light red: A is non-total inhibitor of B.
(0.20 MB TIF)

Text S1 The file contains five supplementary Tables. Table S1 provides a complete list of the network nodes, their used node value levels and abbreviations. Table S2 lists all equations of the model including the according timescale, literature references and organisms of which the information was derived. Table S3 contains the interactions excluded in logical steady state computation and Table S4 lists all non-monotone interactions. Table S5 shows the relative participation of network components in all feedback loops on the respective timescale. At the end of the document all literature references from Table S2 are given in alphabetical order.
(0.33 MB PDF)

Protocol S1 The file contains all experimental data which is not shown in the manuscript.
(0.41 MB PDF)

Protocol S2 The file contains a stepwise manual including screenshots how to install CNA and get started with the model. This very short introduction is added for your convenience. Please note that the CNA download contains a comprehensive manual including much more and detailed information which cannot be replaced by this file.
(0.58 MB PDF)

Protocol S3 The model can be opened with CNA which is a package for MATLAB and is available for free for academic use on http://www.mpi-magdeburg.mpg.de/projects/cna/cna.html. The needed MATLAB license is with costs. The download includes a manual. After starting CNA a new project has to be declared using the given folder ‘ApoptosisModel’ as subdirectory which also includes the network map (apoptosismap.bmp). The textboxes are optimized for width 0.01, height 0.02 and font size 8. To reproduce the logical steady state simulation always first set the default scenario.
(0.09 MB ZIP)
