# Supplementary figures and images for: ON/OFF and Beyond - A Boolean Model of Apoptosis
Source: PLoS Comput Biol. 2009 Dec 11;5(12):e1000595. doi: 10.1371/journal.pcbi.1000595 (PMC2781112; doi:10.1371/journal.pcbi.1000595)

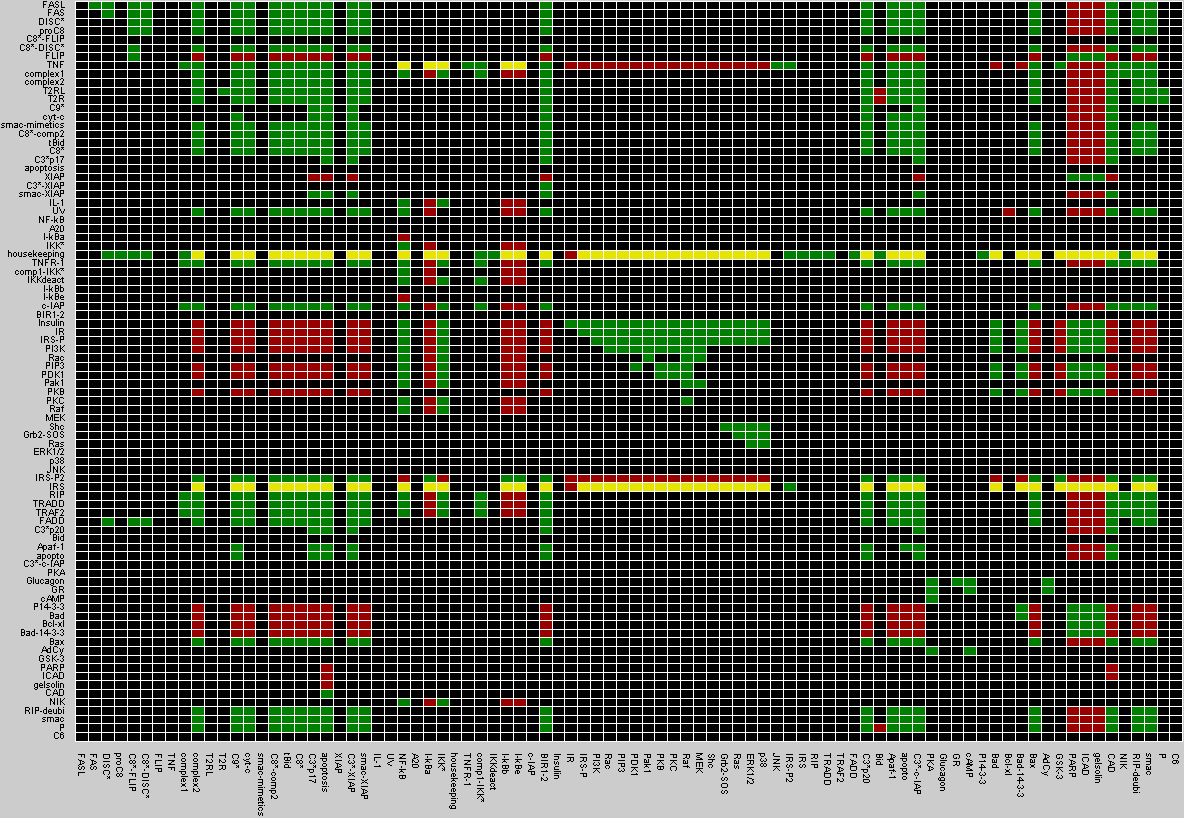

Supplement: Figure S1 — The dependency matrix for τ = 4 displays the influence of each node on each other node in the network. Legend: dark green: A is total activator of B, dark red: A is total inhibitor of B, yellow: A has activating and inhibiting effect on B, black: no influence of A on B, light green: A is non-total activator of B, light red: A is non-total inhibitor of B. (0.18 MB TIF) [file pcbi.1000595.s001.tif]

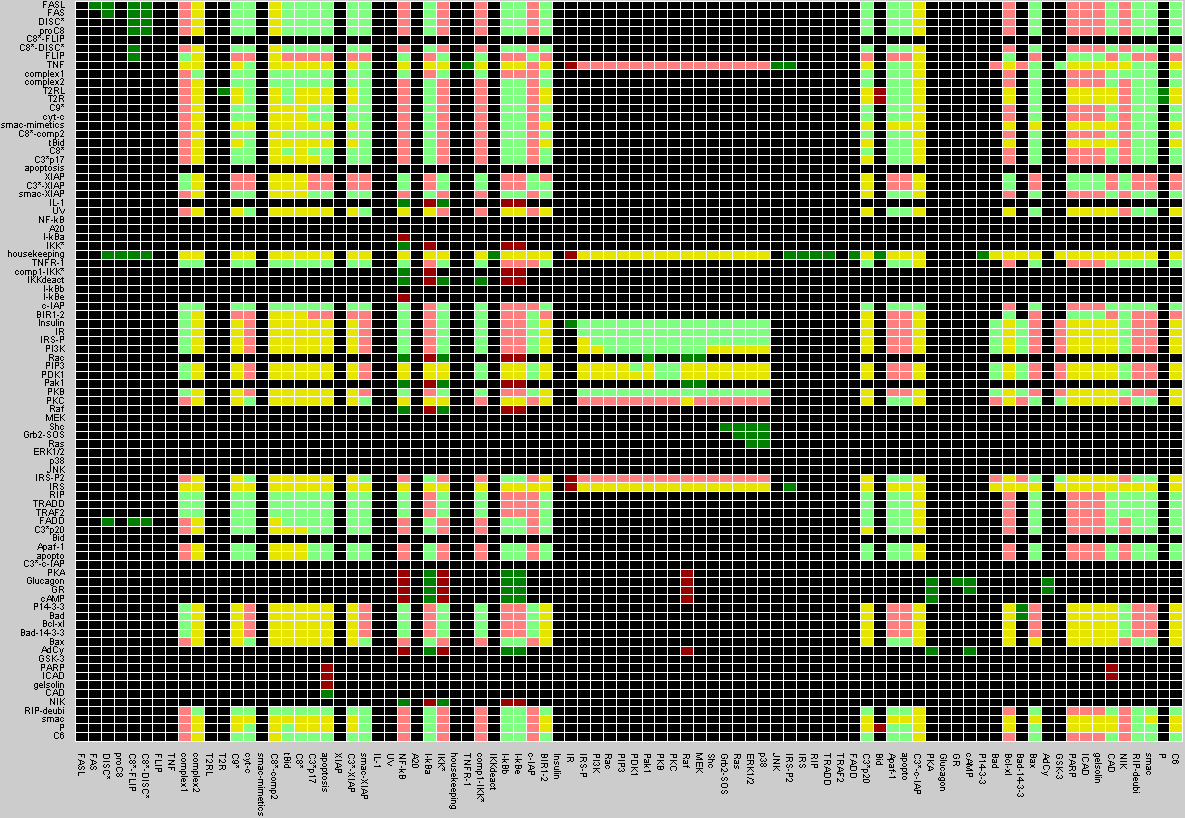

Supplement: Figure S2 — The dependency matrix for τ = 5 displays the influence of each node on each other node in the network. Legend: dark green: A is total activator of B, dark red: A is total inhibitor of B, yellow: A has activating and inhibiting effect on B, black: no influence of A on B, light green: A is non-total activator of B, light red: A is non-total inhibitor of B. (0.19 MB TIF) [file pcbi.1000595.s002.tif]

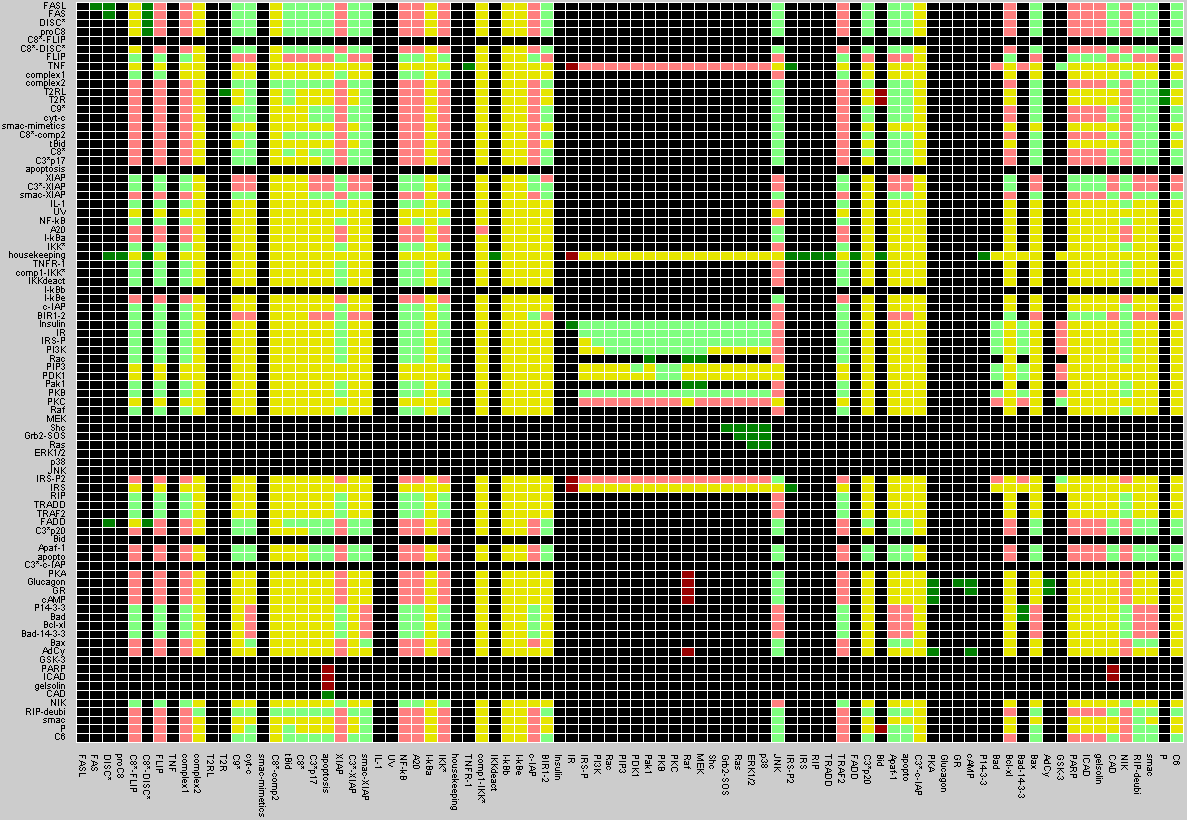

Supplement: Figure S3 — The dependency matrix for τ = 10 displays the influence of each node on each other node in the network. Legend: dark green: A is total activator of B, dark red: A is total inhibitor of B, yellow: A has activating and inhibiting effect on B, black: no influence of A on B, light green: A is non-total activator of B, light red: A is non-total inhibitor of B. (0.20 MB TIF) [file pcbi.1000595.s003.tif]

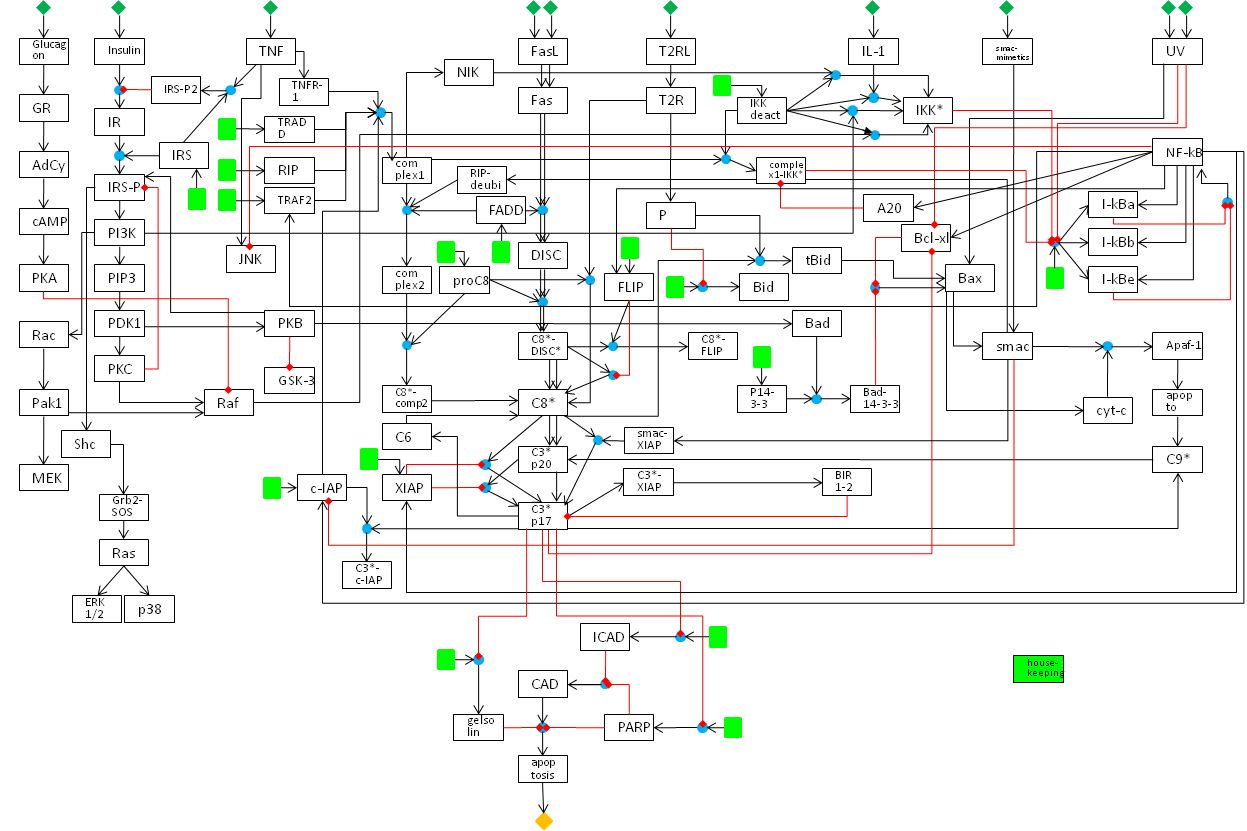

Supplement: Protocol S3 — The model can be opened with CNA which is a package for MATLAB and is available for free for academic use on http://www.mpi-magdeburg.mpg.de/projects/cna/cna.html. The needed MATLAB license is with costs. The download includes a manual. After starting CNA a new project has to be declared using the given folder ‘ApoptosisModel’ as subdirectory which also includes the network map (apoptosismap.bmp). The textboxes are optimized for width 0.01, height 0.02 and font size 8. To reproduce the logical steady state simulation always first set the default scenario. (0.09 MB ZIP) [file pcbi.1000595.s007.zip › ApoptosisModel/apoptosismap.bmp]
