# Supplementary material for: ON/OFF and Beyond - A Boolean Model of Apoptosis
Source: PLoS Comput Biol. 2009 Dec 11;5(12):e1000595. doi: 10.1371/journal.pcbi.1000595 (PMC2781112; doi:10.1371/journal.pcbi.1000595)
Supplement: Text S1 — The file contains five supplementary Tables. Table S1 provides a complete list of the network nodes, their used node value levels and abbreviations. Table S2 lists all equations of the model including the according timescale, literature references and organisms of which the information was derived. Table S3 contains the interactions excluded in logical steady state computation and Table S4 lists all non-monotone interactions. Table S5 shows the relative participation of network components in all feedback loops on the respective timescale. At the end of the document all literature references from Table S2 are given in alphabetical order. (0.33 MB PDF) [file pcbi.1000595.s004.pdf]

Supplementary Tables to ‘ON/OFF and Beyond - a Boolean Model of Apoptosis’

Table of contents

Table S1. Abbreviations, number of used discrete levels and descriptions of the network nodes	2
Table S2. List of all logical equations of the model (inputs/outputs excluded)	4
Table S3. 13 interactions are excluded from logical steady state computation.	8
Table S4. The model contains 14 non-monotone interactions.	9
Table S5. Relative participation of network components in all feedback loops on the respective timescale.	9
Supplementary References	10

Remark on used notation in the Supplementary Tables:

The interactions of the model are given in the notation of the CellNetAnalyzer.

- A logical NOT is represented by “!”.
- A logical AND is represented by “+”.
- The value of a node precedes the name of the node.
- The requirements of an interaction on the left side and its result on the right side are separated by “=”.

Table S1. Abbreviations, number of used discrete levels and descriptions of the network nodes

name of node	levels	trivial name or description
**A20**	1	Inhibitor A20
**AdCy**	1	Adenylate cyclase
**Apaf-1**	1	Apoptotic protease-activating factor 1
**apopto**	1	Apoptosome complex
**apoptosis**	1	Apoptosis output node
**Bad**	1	Bcl-xl/Bcl-2 associated death promoter
**Bad-14-3-3**	1	Heterodimer of Bad and 14-3-3
**Bax**	1	Bcl-2-associated X protein
**Bcl-xl**	1	B-cell lymphoma x-Large
**Bid**	1	Bcl-2 Interacting Domain
**BIR1-2**	1	Baculovirus inhibitory repeats 1 and 2
**C3*-c-IAP**	1	Complex of activated caspase 3 and c-IAP
**C3*p17**	2	P17 form of activated caspase 3
**C3*p20**	2	P20 form of activated caspase 3
**C3*-XIAP**	1	Heterodimer of activated caspase 3 and XIAP
**C6**	1	Caspase 6
**C8***	2	Activated form of caspase 8
**C8*-comp2**	1	Heterodimer of activated caspase 8 and complex2
**C8*-DISC***	2	Heterodimer of activated caspase 8 and the DISC
**C8*-FLIP**	1	Heterodimer of activated caspase 8 and FLIP
**C9***	1	Activated form of caspase 9
**CAD**	1	Caspase activated DNAse
**cAMP**	1	Cyclic adenosine monophosphate
**c-IAP**	2	Cellular inhibitor of apoptosis protein
**comp1**	1	TNFR-1 signaling complex1
**comp1-IKK***	1	Complex of complex1 and activated IKK
**comp2**	1	TNFR-1 signaling complex 2
**cyt-c**	1	Cytochrome c
**DISC***	2	Activated death inducing signalling complex
**ERK1/2**	1	Extracellular signal-regulated kinase 1/2
**FADD**	1	Fas associated death domain
**Fas**	2	Fas receptor
**FasL**	2	Fas ligand
**FLIP**	2	Cellular FLICE inhibitory protein
**Gelsolin**	1	Gelsolin
**Glucagon**	1	Glucagon
**GR**	1	Glucagon receptor
**Grb2-SOS**	1	Heterodimer of growth factor receptor bound protein 2 guanine-nucleotide exchange releasing factor Son of sevenless
**GSK-3**	1	Glycogen synthase kinase 3
**Housekeeping**	1	Housekeeping function
**ICAD**	1	Inhibitor of caspase activated DNAse
**I-kBa**	1	Inhibitor of κB α
**I-kBb**	1	Inhibitor of κB β

**I-kBe**	1	Inhibitor of κB ε
**IKK***	1	Activated form of Ikappa kinase
**IKKdeact**	1	Inactive IKK form
**IL-1**	1	Interleukin-1β
**Insulin**	1	Insulin
**IR**	1	Insulin receptor
**IRS**	1	Unphosphorylated Insulin receptor substrate
**IRS-P**	1	Tyrosine phosphorylated Insulin receptor substrate
**IRS-P2**	1	Serine phosphorylated insulin receptor substrate
**JNK**	1	C-Jun N-terminal kinase
**MEK**	1	MAP-Erk kinase
**NF-κB**	1	Nuclear factor kB
**NIK**	1	NF-κB-inducing kinase
**P**	1	Unknown factor in type 2 apoptosis
**P14-3-3**	1	14-3-3 protein
**p38**	1	*p38* MAP *Kinase*
**Pak1**	1	*P21-Activating Kinase 1*
**PARP**	1	Poly-ADP-ribose polymerase
**PDK1**	1	Pleckstrin homology (PH) domain containing serine protease
**PI3K**	1	Phosphoinositide-3’-OH kinase
**PIP3**	1	Phosphatidylinositol (3,4,5)-trisphosphate
**PKA**	1	Protein kinase B
**PKB**	1	Protein kinase B
**PKC**	1	Protein kinase C
**proC8**	1	Pro-caspase 8
**Rac**	1	Small GTPase Rac
**Raf**	1	Small GTPase Raf-1
**Ras**	1	Small GTPase Ras
**RIP**	1	Receptor associated receptor kinase 1
**RIP-deubi**	1	Deubiquitinated form of RIP
**Shc**	1	Src Homology 2 *Domain* Containing
**Smac**	1	Second mitochondrial derived activator of apoptosis
**smac-mimetics**	1	Smac-mimetics
**smac-XIAP**	1	Heterodimer of smac and XIAP
**T2R**	1	Type 2 receptor
**T2RL**	1	Type 2 receptor ligand
**tBid**	1	Truncated Bid
**TNF**	1	Tumor necrosis factor α
**TNFR-1**	1	TNF receptor 1
**TRADD**	1	TNF receptor associated-protein with death domain
**TRAF2**	1	TNF receptor associated protein 2
**UV**	2	UVB irradiation
**XIAP**	2	X-linked inhibitor of apoptosis

Table S2. List of all logical equations of the model (inputs/outputs excluded)

interaction	timescale τ	reference	organism
**housekeeping = FADD**	0	Harper *et al*, 2003, Muzio *et al*, 1998	*Homo sapiens*
**housekeeping = IKKdeact**	0	Devin *et al*, 2000	*Homo sapiens, Mus musculus*
**housekeeping = IRS**	0	reviewed by White 1998	*Homo sapiens, Mus musculus*
**housekeeping = P14-3-3**	0	Masters *et al*, 2001	*Homo sapiens*
**housekeeping = proC8**	0	Lavrik *et al*, 2003	*Homo sapiens*
**housekeeping = RIP**	0	Wang *et al*, 2008, Devin *et al*, 2000	*Homo sapiens, Mus musculus*
**housekeeping = TRADD**	0	Hsu *et al*, 1995, Hsu *et al*, 1996	*Homo sapiens*
**comp1 + IKKdeact = comp1-IKK***	2	Devin *et al*, 2000	*Homo sapiens, Mus musculus*
**comp1 = NIK**	2	Senftleben *et al*, 2001, Xiao *et al*, 2001	*Homo sapiens, Mus musculus*
**TNF = JNK**	2	Liu *et al*, 1996	*Homo sapiens*
**TNF = TNFR-1**	2	Tartaglia *et al*, 1993	*Homo sapiens*
**TNFR-1 + RIP + TRADD + TRAF-2 + c-IAP = comp1**	2	Boldin *et al*, 1996, Hsu *et al*, 1995, Rothe *et al*, 1995, Mahoney *et al*, 2008	*Homo sapiens, Mus musculus*
**C8*-comp2= C8***	3	Wang *et al*, 2008, Chen and Goeddel, 2002	*Homo sapiens, Mus musculus*
**proC8 + comp2 = C8*-comp2**	3	Chen and Goeddel, 2002	*Homo sapiens, Mus musculus*
**RIP-deubi + comp1 + FADD = comp2**	3	Boldin *et al*, 1996, Wang *et al*, 2008	*Homo sapiens, Mus musculus*
**smac = RIP-deubi**	3	Wang *et al*, 2008	*Mus musculus*
**smac-mimetics = smac**	3	Li *et al*, 2004, Wang *et al*, 2008	*Homo sapiens*
**!BAD-14-3-3 + !Bcl-xl = Bax**	4	Cheng *et al*, 2001	*Mus musculus*
**!I-kBa + !I-kBe = NF-κB**	4	Baeuerle and Baltimore, 1988, Kearns *et al*, 2006	*Homo sapiens, Mus musculus*
**!PARP + !ICAD = CAD**	4	Enari *et al*, 1998, Sakahira *et al*, 1998, reviewed by Cryns and Yuan, 1998	*Homo sapiens, Mus musculus*
**!PKB = GSK-3**	4	Datta *et al*, 1997, reviewed by Nystrom and Quon, 1999	*Homo sapiens, Mus musculus*
**!UV = Bcl-xl**	4	Zhang and Rosdahl, 2005	*Homo sapiens*
**1 C3*p20 = 1 C3*p17**	4	Han *et al*, 1997	*Homo sapiens*
**1 C8* = 1 C3*p20**	4	Han *et al*, 1997	*Homo sapiens*
**1 C8*-DISC* = 1 C8***	4	Lavrik *et al*, 2003	*Homo sapiens*
**1 DISC* + proC8 = 1 C8*-DISC***	4	Lavrik *et al*, 2003	*Homo sapiens*
**1 Fas + FADD =1 DISC***	4	Boldin *et al*, 1996	*Homo sapiens*
**1 FasL = 1 Fas, 2 FasL = 2 Fas**	4	Lavrik *et al*, 2007, Itoh and Nagata, 1993	*Homo sapiens, Mus musculus*
**2 C3*p20 + 2 !XIAP = 2 C3*p17**	4	Deveraux *et al*, 1997, Li *et al*, 2004, Chai *et al*, 2000, Verhagen *et al*, 2000, Rehm *et al*, 2006	*Homo sapiens, Mus musculus*
**2 C8* = 2 C3*p20**	4	Han *et al*, 1997	*Homo sapiens*

**2 C8*-DISC* = 2 C8***	4	Lavrik *et al*, 2003	*Homo sapiens*
**2 DISC* + proC8 = 2 C8*-DISC***	4	Lavrik *et al*, 2003	*Homo sapiens*
**2 FAS + FADD = 2 DISC***	4	Boldin *et al*, 1996	*Homo sapiens*
**AdCy = cAMP**	4	Jelinek *et al*, 1993	*Homo sapiens, Rattus norvegicus*
**Apaf-1 = apopto**	4	Zhou *et al*, 1997, Deveraux *et al*, 1998, Li *et al*, 1997	*Homo sapiens*
**apopto = C9***	4	Zhou *et al*, 1997, Li *et al*, 1997, Slee *et al*, 1999, Srinivasula *et al*, 1998	*Homo sapiens*
**BAD + P14-3-3 = BAD-14-3-3**	4	Datta *et al*, 1997	*Homo sapiens, Mus musculus*
**Bax = cyt-c**	4	Madesh *et al*, 2002, Wang *et al*, 1996, Desagher *et al*, 1999	*Homo sapiens*
**Bax = smac**	4	Madesh *et al*, 2002, Wang *et al*, 1996, Desagher *et al*, 1999	*Homo sapiens, Mus musculus*
**C3*p17 + c-IAP = C3*-c-IAP**	4	Deveraux *et al*, 1997	*Homo sapiens*
**C3*p17 = C3*-XIAP**	4	Deveraux *et al*, 1997	*Homo sapiens*
**C3*-XIAP = BIR1-2**	4	Rehm *et al*, 2006	*Homo sapiens*
**C8* + !XIAP = C3*p17**	4	Li *et al*, 2004, Chai *et al*, 2000, Verhagen *et al*, 2000, Rehm *et al*, 2006	*Homo sapiens, Mus musculus*
**C8* + P = tBid**	4	Zhao *et al*, 2003, Roucou *et al*, 2002	*Mus musculus*
**C8*-DISC* + !FLIP = C8***	4	Lavrik *et al*, 2007, Irmler *et al*, 1997, Tschopp *et al*, 1998	*Homo sapiens, Mus musculus*
**C8*-DISC* + FLIP = C8*-FLIP**	4	Lavrik *et al*, 2007, Irmler *et al*, 1997, Tschopp *et al*, 1998	*Homo sapiens*
**C9* = 2 C3*p20**	4	Slee *et al*, 1999	*Homo sapiens*
**CAD + !PARP + !gelsolin = apoptosis**	4	Enari *et al*, 1998, Sakahira *et al*, 1998, reviewed by Cryns and Yuan 1998	*Homo sapiens, Mus musculus*
**cAMP = PKA**	4	Cheng *et al*, 1998	*Homo sapiens, Mus musculus*
**cyt-c + smac = Apaf-1**	4	Zhou *et al*, 1997	*Homo sapiens*
**Glucagon = GR**	4	Jelinek *et al*, 1993	*Homo sapiens, Rattus norvegicus*
**GR = AdCy**	4	Jelinek *et al*, 1993	*Homo sapiens, Rattus norvegicus*
**Grb2-SOS = Ras**	4	reviewed by Kolch 2000	*Homo sapiens, Mus musculus*
**housekeeping + !C3*p17 = gelsolin**	4	Kothakota *et al*, 1997, Janicke *et al*, 1998	*Homo sapiens, Mus musculus*
**housekeeping + !IKK* + !comp1-IKK* + 2 !UV = I-kBa**	4	Mercurio *et al*, 1997, DiDonato *et al*, 1997	*Homo sapiens*
**housekeeping + !IKK* + !comp1-IKK* + 2 !UV = I-kBb**	4	Mercurio *et al*, 1997, DiDonato *et al*, 1997	*Homo sapiens*

**housekeeping + !IKK* + !comp1-IKK* + 2 !UV = I-kBe**	4	Mercurio *et al*, 1997, DiDonato *et al*, 1997	*Homo sapiens*
**housekeeping + !P = Bid**	4	McKenzie *et al*, 2008, Krams *et al*, 2007	*Mus musculus*
**housekeeping + 2 !C3*p17 = PARP**	4	Enari *et al*, 1998, Lazebnik *et al*, 1994, Sakahira *et al*, 1998	*Homo sapiens, Mus musculus*
**housekeeping + 2!C3*p17 = ICAD**	4	Kothakota *et al*, 1997, Janicke *et al*, 1998	*Homo sapiens, Mus musculus*
**IL-1 + IKKdeact = IKK***	4	Poeppelmann *et al*, 2005, Barisic *et al*, 2008, Kothny-Wilkes *et al*, 1999	*Homo sapiens*
**Insulin + !IRS-P2 = IR**	4	reviewed by Gual *et al*, 2005	*Homo sapiens, Mus musculus*
**IR + IRS = IRS-P**	4	reviewed by White 1998, Saltiel and Kahn, 2001, reviewed by Nystrom and Quon, 1999, reviewed by White 2002	*Homo sapiens, Mus musculus*
**IRS-P = PI3K**	4	Saltiel and Kahn, 2001, reviewed by Nystrom and Quon, 1999, reviewed by White 2002	*Homo sapiens, Mus musculus*
**IRS-P = Shc**	4	Saltiel and Kahn, 2001, reviewed by White 2002	*Homo sapiens, Mus musculus*
**NIK + IKKdeact = IKK***	4	Senftleben *et al*, 2001, Xiao *et al*, 2001	*Homo sapiens, Mus musculus*
**Pak1 = MEK**	4	reviewed by Kolch 2000	*Homo sapiens, Mus musculus*
**Pak1 = Raf**	4	reviewed by Kolch 2000	*Homo sapiens, Mus musculus*
**PDK1 = PKB**	4	Good *et al*, 1998, Vanhaesebrock *et al*, 2000	*Homo sapiens*
**PDK1 = PKC**	4	Pruett *et al*, 1995, Good *et al*, 1998	*Homo sapiens*
**PI3K + IKKdeact = IKK***	4	Datta *et al*, 1997	*Homo sapiens, Mus musculus*
**PI3K = PIP3**	4	Saltiel and Kahn, 2001, reviewed by Nystrom and Quon, 1999, reviewed by White 2002	*Homo sapiens, Mus musculus*
**PI3K = Rac**	4	Saltiel and Kahn, 2001, reviewed by Nystrom and Quon, 1999, reviewed by White 2002	*Homo sapiens, Mus musculus*
**PIP3 = PDK1**	4	Vanhaesebrock *et al*, 2000, Saltiel and Kahn, 2001, reviewed by Nystrom and Quon, 1999, reviewed by White 2002	*Homo sapiens, Mus musculus*
**PKB = BAD**	4	Datta *et al*, 1997	*Homo sapiens, Mus musculus*
**PKC = Raf**	4	reviewed by Kolch 2000	*Homo sapiens, Mus musculus*
**Rac = Pak1**	4	Saltiel and Kahn, 2001, reviewed by Nystrom and Quon, 1999, reviewed by White 2002	*Homo sapiens, Mus musculus*
**Raf + IKKdeact = IKK***	4	Lin *et al*, 1999	*Homo sapiens*

**Ras = ERK1/2**	4	reviewed by Kolch 2000	*Homo sapiens, Mus musculus*
**Ras = p38**	4	reviewed by Kolch 2000	*Homo sapiens, Mus musculus*
**Shc = Grb2-SOS**	4	Pruett *et al*, 1995, Saltiel and Kahn, 2001, reviewed by White 2002	*Homo sapiens, Mus musculus*
**smac = smac-XIAP**	4	Li *et al*, 2004, Chai *et al*, 2000, Verhagen *et al*, 2000, Rehm *et al*, 2006	*Homo sapiens, Mus musculus*
**smac-XIAP + 1 C8* = 2 C3*p17**	4	Li *et al*, 2004, Chai *et al*, 2000, Verhagen *et al*, 2000, Rehm *et al*, 2006	*Homo sapiens, Mus musculus*
**T2R + C8 = C8***	4	based on Scaffidi *et al*, 1998, Walter *et al*, 2008	*Homo sapiens, Mus musculus*
**T2R = P**	4	based on Scaffidi *et al*, 1998, Walter *et al*, 2008	*Homo sapiens, Mus musculus*
**T2RL = T2R**	4	based on Scaffidi *et al*, 1998, Walter *et al*, 2008	*Homo sapiens, Mus musculus*
**tBid = Bax**	4	Wei *et al*, 2001, Madesh *et al*, 2002, Wang *et al*, 1996, Desagher *et al*, 1999, Zhao *et al*, 2003	*Homo sapiens, Mus musculus*
**TNF + IRS = IRS-P2**	4	Gual *et al*, 2005	*Homo sapiens, Mus musculus*
**UV = Bax**	4	Chen *et al*, 2007	*Mus musculus*
**!BIR1-2 = C3*p17**	5	Rehm *et al*, 2006, Deveraux *et al*, 1997	*Homo sapiens*
**!C3*p17 = Bcl-xl**	5	Cheng *et al*, 1998, Clem *et al*, 1998	*Homo sapiens, Rattus norvegicus*
**!PKA = Raf**	5	reviewed by Kolch 2000	*Homo sapiens, Mus musculus*
**!PKC = IRS-P**	5	Fea and Roth, 1997, Ravichandran *et al*, 2001	*Homo sapiens, Mus musculus*
**!smac = c-IAP**	5	Wang *et al*, 2008	*Homo sapiens*
**2 C3*p17 = C9***	5	Zhou *et al*, 2003, Rehm *et al*, 2006, Deveraux *et al*, 1997	*Homo sapiens*
**C3*p17 = C6**	5	Cowling and Downward 2002, Murphy *et al*, 2004	*Homo sapiens*
**C6 = C8***	5	Murphy *et al*, 2004	*Homo sapiens*
**PKB = IRS-P**	5	Paz *et al*, 1999, reviewed by Nystrom and Quon, 1999	*Homo sapiens, Rattus norvegicus*
**!A20 = comp1-IKK***	10	Song *et al*, 1996, Wertz *et al*, 2004, Lee *et al*,2000	*Homo sapiens, Mus musculus*
**!NF-κB = JNK**	10	Tang *et al*, 2001	*Mus musculus*
**housekeeping = c-IAP**	10	Poeppelmann *et al*, 2005, Barisic *et al*, 2008	*Homo sapiens*
**housekeeping = FLIP**	10	Poeppelmann *et al*, 2005	*Homo sapiens*
**housekeeping = XIAP**	10	Poeppelmann *et al*, 2005	*Homo sapiens*
**housekeeping = TRAF-2**	10	Krikos *et al*, 1992	*Homo sapiens*
**NF-κB = 2 XIAP**	10	Barisic *et al*, 2008	*Homo sapiens*
**NF-κB = 2 c-IAP**	10	Barisic *et al*, 2008	*Homo sapiens*

**NF-κB = 2 FLIP**	10	Kreuz *et al*, 2001, Micheau *et al*, 2001, Barisic *et al*, 2008	*Homo sapiens, Mus musculus*
**NF-κB = A20**	10	Song *et al*, 1996, Krikos *et al*, 1992	*Homo sapiens, Mus musculus*
**NF-κB = Bcl-xl**	10	Lee *et al*, 1999, Tamatani *et al*, 1999, Chen *et al*, 2000	*Homo sapiens, Rattus norvegicus*
**NF-κB = I-kBa**	10	Tian *et al*, 2005	*Homo sapiens*
**NF-κB = I-kBb**	10	Tian *et al*, 2005	*Homo sapiens*
**NF-κB = I-kBe**	10	Rothe *et al*, 1995, Wang *et al*, 1998	*Homo sapiens, Mus musculus*
**NF-κB = TRAF-2**	10	Poeppelmann *et al*, 2005, Barisic *et al*, 2008, Kothny-Wilkes *et al*, 1999	*Homo sapiens*

Table S3. 13 interactions are excluded from logical steady state computation.

no.	interaction
1	**!A20 = comp1-IKK***
2	**!BIR1-2 = C3*p17**
3	**!NF-κB = JNK**
4	**!PKA = Raf**
5	**!PKC = IRS-P**
6	**!smac = c-IAP**
7	**2 C3*p17 = C9***
8	**C3*p17 = C6**
9	**C6 = C8***
10	**NF-κB = I-kBa**
11	**NF-κB = I-kBb**
12	**NF-κB = I-kBe**
13	**PKB = IRS-P**

Table S4. The model contains 14 non-monotone interactions.

**1 C3*p20 = 1 C3*p17**
**1 C8* = 1 C3*p20**
**1 C8*-DISC* = 1 C8***
**1 DISC* + proC8 = 1 C8*-DISC***
**1 FAS + FADD = 1 DISC***
**1 FASL = 1 FAS**
**2 C3*p17 = C9***
**2 C3*p20 + 2 !XIAP = 2 C3*p17**
**2 C8* = 2 C3*p20**
**2 C8*-DISC* = 2 C8***
**2 DISC* + proC8 = 2 C8*-DISC***
**2 FAS + FADD = 2 DISC***
**2 FASL = 2 FAS**
**smac-XIAP + 1 C8* = 2 C3*p17**

Table S5. Relative participation of network components in all feedback loops on the respective timescale.

**τ = 5**			**τ = 10**		
positive & negative feedback loops	positive feed-back loops	negative feed-back loops	positive & negative feedback loops	positive feed-back loops	negative feed-back loops
C3*p17 (89 %)<br>C8 (71 %)<br>Bax (69 %)<br>C3*p20 (63 %)<br>smac (57 %)<br>Bcl-xl (49 %)<br>complex2, C8*-comp2 (40 %)<br>C6 (31 %)<br>C9* (29 %)<br>apopto, Apaf-1 (23 %)<br>tBid, RIP-deubi, complex1, c-IAP (20 %)<br>cyt-c (11 %)<br>smac-XIAP, PIP3, PI3K, PDK1, IRS-P (6 %)<br>PKC, PKB, C3*-XIAP, BIR1-2 (3 %)	C3*p17 (92 %)<br>C8, C3*p20 (69 %)<br>Bax (65 %)<br>smac (50 %)<br>C6, Bcl-xl (42 %)<br>C9* (38 %)<br>Apaf-1, apopto (31 %)<br>RIP-deubi, C8*-comp2, complex2 (27%)<br>tBid (23 %)<br>cyt-c (15 %)<br>smac-XIAP (8 %)<br>IRS-P, PI3K, PIP3, PDK1, PKB (4 %)	Bax, smac, complex1, complex2, C8*-comp2, C8, C3*p17, c-IAP (78 %)<br>Bcl-xl (67 %)<br>C3*p20 (44 %)<br>tBid, C3*-XIAP, BIR1-2, IRS-P, PI3K, PIP3, PDK1, PKC (11 %)	Bax (76 %)<br>C3*p17 (75 %)<br>smac (72 %)<br>complex1 (66 %)<br>C8* (64 %)<br>NF-κB (63 %)<br>c-IAP (62 %)<br>Bcl-xl (56 %)<br>C3*p20 (40 %)<br>I-κBε, I-κBα, comp1-IKK* (32 %)<br>NIK, FLIP, IKK* (29 %)<br>C6, tBid (20 %)<br>XIAP (17 %)<br>complex2, C8*-comp2 (15 %)<br>C9* (11 %)<br>Apaf-1, apopto (8 %)<br>RIP-deubi (7 %)<br>TRAF2, cyt-c (4 %)<br>IRS-P, PI3K, PIP3, PDK1, A20, smac-XIAP (2 %)<br>C3*-XIAP, BIR1-2, PKB, PKC (1 %)	Bax (79 %)<br>C3*p17 (78 %)<br>smac (74 %)<br>complex1, NF-κB (68 %)<br>C8* (66 %)<br>c-IAP (63 %)<br>Bcl-xl (57 %)<br>C3*p20 (41 %)<br>FLIP, I-κBα, IKK*, comp1-IKK*, I-κBε, NIK (34 %)<br>C6 (23 %)<br>tBid (22 %)<br>XIAP (20 %)<br>C9* (12 %)<br>Apaf-1, apopto (10 %)<br>complex2, C8*-comp2, RIP-deubi (9 %)<br>cyt-c, TRAF2 (5 %)<br>smac-XIAP (2 %)<br>IRS-P, PI3K, PIP3, PDK1, PKB (1 %)	complex1, complex2, C8*-comp2, C8*, C3*p17, c-IAP, Bax, smac (54 %)<br>Bcl-xl (46 %)<br>NF-κB, C3*p20 (31 %)<br>A20, I-κBα, comp1-IKK*, I-κBε (15 %)<br>tBid, C3*-XIAP, BIR1-2, IRS-P, PI3K, PIP3, PDK1, PKC (8 %)

Supplementary References

Baeuerle PA, Baltimore D (1988) I kappa B: a specific inhibitor of the NF-kappa B transcription factor. Science 242: 540-546.

Barisic S, Strozyk E, Peters N, Walczak H, Kulms D (2008) Identification of PP2A as a crucial regulator of the NF-[kappa]B feedback loop: its inhibition by UVB turns NF-[kappa]B into a pro-apoptotic factor. Cell Death Differ: 1476-5403.

Boldin MP, Goncharov TM, Goltseve YV, Wallach D (1996) Involvement of MACH, a novel MORT1/FADD-interacting protease, in Fas/APO-1- and TNF receptor-induced cell death. Cell 85: 803-815.

Chai J, Du C, Wu JW, Kyin S, Wang X, et al. (2000) Structural and biochemical basis of apoptotic activation by Smac/DIABLO. Nature 406: 855-862.

Chen C, Edelstein LC, Gelinas C (2000) The Rel/NF-kappaB family directly activates expression of the apoptosis inhibitor Bcl-x(L). Mol Cell Biol 20: 2687-2695.

Chen G, Goeddel DV (2002) TNF-R1 signaling: a beautiful pathway. Science, 296(5573), 1634-1635.

Chen M, Xing D, Chen T, Zhang L (2007) BimL involvement in Bax activation during UV irradiation-induced apoptosis. Biochem Biophys Res Commun 358: 559-65.

Cheng X, Ma Y, Moore M, Hemmings BA, Taylor SS (1998) Phosphorylation and activation of cAMP-dependent protein kinase by phosphoinositide-dependent protein kinase. Proc Natl Acad Sci USA 95: 9849-9854.

Cheng EH, Wei MC, Weiler S, Flavell RA, Mak TW, et al. (2001) BCL-2, BCL-X(L) sequester BH3 domain-only molecules preventing BAX- and BAK-mediated mitochondrial apoptosis. Molecular Cell 8: 705-711.

Clem RJ, Cheng EH, Karp CL, Kirsch DG, Ueno K, et al. (1998) Modulation of cell death by Bcl-x L through caspase interaction. Proc Natl Acad Sci USA 95: 554-559.

Cowling V, Downward J (2002) Caspase-6 is the direct activator of caspase-8 in the cytochrome c-induced apoptosis pathway: absolute requirement for removal of caspase-6 prodomain. Cell Death Differ 9: 1046-1056.

Cryns V, Yuan J (1998) Proteases to die for. Genes Dev 12: 1551-1570.

Datta SR, Dudek H, Tao X, Masters S, Fu H, et al. (1997) Akt phosphorylation of BAD couples survival signals to the cell-intrinsic death machinery. Cell 91: 231-241.

Desagher S, Osen-Sand A, Nichols A, Eskes R, Montessuit S, et al. (1999) Bid-induced conformational change of Bax is responsible for mitochondrial cytochrome c release during apoptosis. J Cell Biol 144: 891-901.

Deveraux QL, Takahashi R, Salvesen GS, Reed JC (1997) X-linked IAP is a direct inhibitor of cell-death proteases. Nature 388: 300-304.

Deveraux QL, Roy N, Stennicke HR, Van Arsdale T, Zhou Q, et al. (1998) IAPs block apoptotic events induced by caspase-8 and cytochrome c by direct inhibition of distinct caspases. EMBO J 17: 2215-2223.

Devin A, Cook A, Lin Y, Rodriguez Y, Kelliher M, et al. (2000) The distinct roles of TRAF2 and RIP in IKK activation by TNF-R1: TRAF2 recruits IKK to TNF-R1 while RIP mediates IKK activation. Immunity 12: 419-429.

DiDonato JA, Hayakawa M, Rothwarf DM, Zandi E, Karin M (1997) A cytokine-responsive I[kappa]B kinase that activates the transcription factor NF-[kappa]B. Nature 388: 548-554.

Enari M, Sakahira H, Yokoyama H, Okawa K, Iwamatsu A, Nagata S (1998) A caspase-activated DNase that degrades DNA during apoptosis, and its inhibitor ICAD. Nature 391: 43-50.

Fea K De, Roth RA (1997) Protein kinase C modulation of insulin receptor substrate-1 tyrosine phosphorylation requires serine 612. Biochemistry 36: 12939-12947.

Good JA Le, Ziegler WH, Parekh DB, Alessi DR, Cohen P, et al. (1998) Protein kinase C isotypes controlled by phosphoinositide 3-kinase through the protein kinase PDK1. Science 281: 2042-2045.

Gual P, Marchand-Brustel Y Le, Tanti JF (2005) Positive and negative regulation of insulin signaling through IRS-1 phosphorylation. Biochimie 87: 99-109.

Han Z, Hendrickson EA, Bremner TA, Wyche JH (1997) A sequential two-step mechanism for the production of the mature p17:p12 form of caspase-3 in vitro. J Biol Chem 272: 13432-13436.

Harper N, Hughes M, MacFarlane M, Cohen GM (2003) Fas-associated death domain protein and caspase-8 are not recruited to the tumor necrosis factor receptor 1 signaling complex during tumor necrosis factor-induced apoptosis. J Biol Chem 278: 25534-25541.

Hsu H, Xiong J, Goeddel DV (1995) The TNF receptor 1-associated protein TRADD signals cell death and NF-kB activation. Cell 81: 495-504.

Hsu H, Shu HB, Pan MG, Goeddel DV (1996) TRADD-TRAF2 and TRADD-FADD interactions define two distinct TNF receptor 1 signal transduction pathways. Cell 84: 299-308.

Irmler M, Thome M, Hahne M, Schneider P, Hofmann K, et al. (1997) Inhibition of death receptor signals by cellular FLIP. Nature 388: 190-195.

Itoh N, Nagata S (1993) A novel protein domain required for apoptosis. Mutational analysis of human Fas antigen. J Biol Chem 268: 10932-10937.

Janicke RU, Sprengart ML, Wati MR, Porter AG (1998) Caspase-3 is required for DNA fragmentation and morphological changes associated with apoptosis. J Biol Chem 273: 9357-9360.

Jelinek LJ, Lok S, Rosenberg GB, Smith RA, Grant FJ, et al. (1993) Expression cloning and signaling properties of the rat glucagon receptor. Science 259: 1614-1616.

Kearns JD, Basak S, Werner SL, Huang CS, Hoffmann A (2006) IkappaBepsilon provides negative feedback to control NF-kappaB oscillations, signaling dynamics, and inflammatory gene expression. J Cell Biol 173: 659-664.

Kolch W (2000) Meaningful relationships: the regulation of the Ras/Raf/MEK/ERK pathway by protein interactions. Biochem J 351: 289-305.

Kothakota S, Azuma T, Reinhard C, Klippel A, Tang J, et al. (1997) Caspase-3-generated fragment of gelsolin: effector of morphological change in apoptosis. Science 278: 294-298.

Kothny-Wilkes G, Kulms D, Luger TA, Kubin M, Schwarz T (1999) Interleukin-1 protects transformed keratinocytes from tumor necrosis factor-related apoptosis-inducing ligand- and CD95-induced apoptosis but not from ultraviolet radiation-induced apoptosis. J Biol Chem 274: 28916-28921.

Krams SM, Nagasaki K, Esquivel CO, Martinez OM, Riddle E (2007) Mutations to Bid cleavage sites protect hepatocytes from apoptosis following ischemia/reperfusion injury. J Immunol 178: 208-209.

Kreuz S, Siegmund D, Scheurich P, Wajant H (2001) NF-kappaB inducers upregulate cFLIP, a cycloheximide-sensitive inhibitor of death receptor signaling. Mol Cell Biol 21: 3964-3973.

Krikos A, Laherty CD, Dixit VM (1992) Transcriptional activation of the tumor necrosis factor alpha-inducible zinc finger protein, A20, is mediated by kappa B elements. J Biol Chem 267: 17971-17976.

Lavrik I, Krueger A, Schmitz I, Baumann S, Weyd H, et al. (2003) The active caspase-8 heterotetramer is formed at the CD95 DISC. Cell Death Differ 10: 144-145.

Lavrik IN, Golks A, Riess D, Bentele M, Eils R, et al. (2007) Analysis of CD95 threshold signaling: triggering of CD95 (FAS/APO-1) at low concentrations primarily results in survival signaling. J Biol Chem 282: 13664-13671.

Lazebnik YA, Kaufmann SH, Desnoyers S, Poirier GG, Earnshaw WC (1994) Cleavage of poly(ADP-ribose) polymerase by a proteinase with properties like ICE. Nature 371: 346-347.

Lee HH, Dadgostar H, Cheng Q, Shu J, Cheng G (1999) NF-kB-mediated up-regulation of Bcl-x and Bfl-1/A1 is required for CD40 survival signaling in B lymphocytes. Proc Natl Acad Sci USA 96: 9136-9141.

Lee EG, Boone DL, Chai S, Libby SL, Chien M, et al. (2000) Failure to regulate TNF-induced NF-kappaB and cell death responses in A20-deficient mice. Science 289: 2350-2354.

Li P, Nijhawan D, Budihardjo I, Srinivasula SM, Ahmad M, et al. (1997) Cytochrome c and dATP-dependent formation of Apaf-1/caspase-9 complex initiates an apoptotic protease cascade. Cell 91: 479-489.

Li L, Thomas RM, Suzuki H, De Brabander JK, Wang X, et al. (2004) A small molecule Smac mimic potentiates TRAIL- and TNFalpha-mediated cell death. Science 305: 1471-1474.

Lin X, Cunningham ET, Mu Y, Geleziunas R, Greene WC (1999) The proto-oncogene Cot kinase participates in CD3/CD28 induction of NF-kappaB acting through the NF-kappaB-inducing kinase and IkappaB kinases. Immunity 10: 271-280.

Liu ZG, Hsu H, Goeddel DV, Karin M (1996) Dissection of TNF receptor 1 effector functions: JNK activation is not linked to apoptosis while NF-kappaB activation prevents cell death. Cell 87: 565-576.

Madesh M, Antonsson B, Srinivasula SM, Alnemri ES, Hajnóczky G (2002) Rapid kinetics of tBid-induced cytochrome c and Smac/DIABLO release and mitochondrial depolarization. J Biol Chem 277: 5651-5659.

Mahoney DJ, Cheung HH, Mrad RL, Plenchette S, Simard C, et al. (2008) Both cIAP1 and cIAP2 regulate TNFalpha-mediated NF-kappaB activation. Proc Natl Acad Sci USA 105: 11778-11783.

Masters SC, Yang H, Datta SR, Greenberg ME, Fu H (2001) 14-3-3 inhibits Bad-induced cell death through interaction with serine-136. Mol Pharmacol 60: 1325-1331.

McKenzie MD, Carrington EM, Kaufmann T, Strasser A, Huang DCS, et al. (2008) Proapoptotic BH3-only protein Bid is essential for death receptor-induced apoptosis of pancreatic beta-cells. Diabetes 57: 1284-1292.

Mercurio F, Zhu H, Murray BW, Shevchenko A, Bennett BL, et al. (1997) IKK-1 and IKK-2: cytokine-activated IkappaB kinases essential for NF-kappaB activation. Science 278: 860-866.

Micheau O, Lens S, Gaide O, Alevizopoulos K, Tschopp J (2001) NF-kappaB signals induce the expression of c-FLIP. Mol Cell Biol 21: 5299-5305.

Murphy BM, Creagh EM, Martin SJ (2004) Interchain proteolysis, in the absence of a dimerization stimulus, can initiate apoptosis-associated caspase-8 activation. J Biol Chem 279: 36916-36922.

Muzio M, Stockwell BR, Stennicke HR, Salvesen GS, Dixit VM (1998) An induced proximity model for caspase-8 activation. J Biol Chem 273: 2926-2930.

Nystrom FH, Quon MJ (1999) Insulin signalling: metabolic pathways and mechanisms for specificity. Cellular Signalling 11: 563-574.

Paz K, Liu YF, Shorer H, Hemi R, LeRoith D, et al. (1999) Phosphorylation of insulin receptor substrate-1 (IRS-1) by protein kinase B positively regulates IRS-1 function. J Biol Chem 274: 28816-28822.

Pöppelmann B, Klimmek K, Strozyk E, Voss R, Schwarz T, et al. (2005) NFkappaB-dependent down-regulation of tumor necrosis factor receptor-associated proteins contributes to interleukin-1-mediated enhancement of ultraviolet B-induced apoptosis. J Biol Chem 280: 15635-15643.

Pruett W, Yuan Y, Rose E, Batzer AG, Harada N, et al. (1995) Association between GRB2/Sos and insulin receptor substrate 1 is not sufficient for activation of extracellular signal-regulated kinases by interleukin-4: implications for Ras activation by insulin. Mol Cell Biol 15: 1778-1785.

Ravichandran LV, Chen H, Li Y, Quon MJ (2001) Phosphorylation of PTP1B at Ser(50) by Akt impairs its ability to dephosphorylate the insulin receptor. Mol. Endocrinol 15: 1768-1780.

Rehm M, Huber HJ, Dussmann H, Prehn JHM (2006) Systems analysis of effector caspase activation and its control by X-linked inhibitor of apoptosis protein. EMBO J 25: 4338-4349.

Rothe M, Pan MG, Henzel WJ, Ayres TM, Goeddel DV (1995) The TNFR2-TRAF signaling complex contains two novel proteins related to baculoviral inhibitor of apoptosis proteins. Cell 83: 1243-1252.

Roucou X, Montessuit S, Antonsson B, Martinou JC (2002) Bax oligomerization in mitochondrial membranes requires tBid (caspase-8-cleaved Bid) and a mitochondrial protein. Biochem J 368: 915-921.

Sakahira H, Enari M, Nagata S (1998) Cleavage of CAD inhibitor in CAD activation and DNA degradation during apoptosis. Nature 391: 96-99.

Saltiel AR, Kahn CR (2001) Insulin signalling and the regulation of glucose and lipid metabolism. Nature 414: 799-806.

Scaffidi C, Fulda S, Srinivasan A, Friesen C, Li F, et al. (1998) Two CD95 (APO-1/Fas) signaling pathways. EMBO J 17: 1675-1687.

Senftleben U, Cao Y, Xiao G, Greten FR, Krahn G, et al. (2001) Activation by IKKalpha of a second, evolutionary conserved, NF-kappa B signaling pathway. Science 293: 1495-1499.

Slee EA, Harte MT, Kluck RM, Wolf BB, Casiano CA, et al. (1999) Ordering the cytochrome c-initiated caspase cascade: hierarchical activation of caspases-2, -3, -6, -7, -8, and -10 in a caspase-9-dependent manner. J Cell Biol 144: 281-292.

Song HY, Rothe M, Goeddel DV (1996) The tumor necrosis factor-inducible zinc finger protein A20 interacts with TRAF1/TRAF2 and inhibits NF-kappaB activation. Proc Natl Acad Sci USA 93: 6721-6725.

Srinivasula SM, Ahmad M, Fernandes-Alnemri T, Alnemri ES (1998) Autoactivation of procaspase-9 by Apaf-1-mediated oligomerization. Mol Cell 1: 949-957.

Tamatani M, Che YH, Matsuzaki H, Ogawa S, Okado H, et al. (1999) Tumor necrosis factor induces Bcl-2 and Bcl-x expression through NFkappaB activation in primary hippocampal neurons. J Biol Chem 274: 8531-8538.

Tang G, Minemoto Y, Dibling B, Purcell NH, Li Z, et al. (2001) Inhibition of JNK activation through NF-[kappa]B target genes. Nature 414: 313-317.

Tartaglia LA, Rothe M, Hu YF, Goeddel DV (1993) Tumor necrosis factor's cytotoxic activity is signaled by the p55 TNF receptor. Cell 73: 213-216.

Tian B, Nowak DE, Jamaluddin M, Wang S, Brasier AR (2005) Identification of direct genomic targets downstream of the nuclear factor-kappaB transcription factor mediating tumor necrosis factor signaling. J Biol Chem 280: 17435-17448.

Tschopp J, Irmler M, Thome M (1998) Inhibition of Fas death signals by FLIPs. Current Opinion in Immunology 10: 552-558.

Vanhaesebroeck B, Alessi DR (2000) The PI3K-PDK1 connection: more than just a road to PKB. Biochem J 346: 561-576.

Verhagen AM, Ekert PG, Pakusch M, Silke J, Connolly LM, et al. (2000) Identification of DIABLO, a mammalian protein that promotes apoptosis by binding to and antagonizing IAP proteins. Cell 102: 43-53.

Walter D, Schmich K, Vogel S, Pick R, Kaufmann T, et al. (2008) Switch from type II to I Fas/CD95 death signaling on in vitro culturing of primary hepatocytes. Hepatology 48: 1942-1953.

Wang K, Yin XM, Chao DT, Milliman CL, Korsmeyer SJ (1996) BID: a novel BH3 domain-only death agonist. Genes Dev 10: 2859-2869.

Wang CY, Mayo MW, Korneluk RG, Goeddel DV, Baldwin AS Jr. (1998) NF-kappaB antiapoptosis: induction of TRAF1 and TRAF2 and c-IAP1 and c-IAP2 to suppress caspase-8 activation. Science 281: 1680-1683.

Wang L, Du F, Wang X (2008) TNF-alpha induces two distinct caspase-8 activation pathways. Cell 133: 693-703.

Wei MC, Zong WX, Cheng EHY, Lindsten T, Panoutsakopoulou V, et al. (2001) Proapoptotic BAX and BAK: a requisite gateway to mitochondrial dysfunction and death. Science 292: 727-730.

Wertz IE, O'Rourke KM, Zhou H, Eby M, Aravind L, et al. (2004) De-ubiquitination and ubiquitin ligase domains of A20 downregulate NF-[kappa]B signalling. Nature 430: 694-699.

White MF (1998) The IRS-signalling system: A network of docking proteins that mediate insulin action. Molecular and Cellular Biochemistry 182: 3-11.

White MF (2002) IRS proteins and the common path to diabetes. Am J Physiol Endocrinol Metab 283: 413-422.

Xiao G, Harhaj EW, Sun SC (2001) NF-kappaB-inducing kinase regulates the processing of NF-kappaB2 p100. Mol Cell 7: 401-409.

Zhang H, Rosdahl I (2005) Bcl-xL and bcl-2 proteins in melanoma progression and UVB-induced apoptosis. Int J Oncol 28: 661-666.

Zhao Y, Ding W, Qian T, Watkins S, Lemasters JJ, et al. (2003) Bid activates multiple mitochondrial apoptotic mechanisms in primary hepatocytes after death receptor engagement. Gastroenterology 125: 854-867.

Zhou H, Henzel WJ, Liu XG, Lutschg A, Wang X (1997) Apaf-1, a human protein homologous to C. elegans CED-4, participates in cytochrome c-dependent activation of caspase-3. Cell 90: 405-413.
