# Supplementary material for: ON/OFF and Beyond - A Boolean Model of Apoptosis
Source: PLoS Comput Biol. 2009 Dec 11;5(12):e1000595. doi: 10.1371/journal.pcbi.1000595 (PMC2781112; doi:10.1371/journal.pcbi.1000595)
Supplement: Protocol S1 — The file contains all experimental data which is not shown in the manuscript. (0.41 MB PDF) [file pcbi.1000595.s005.pdf]

Supplementary Experiments to 'ON/OFF and Beyond - a Boolean Model of Apoptosis'

Table of contents

NF-κB-DNA binding in response to FasL treatment in Jurkat T cells	2
IκB-α Western Blot in hepatocytes and Jurkat T cells	3
cFLIP and cIAP2 mRNA levels in Jurkat T cells	4
XIAP Western Blot in hepatocytes and Jurkat T cells	5
Caspase-3 activity in response to FasL and UV irradiation in Jurkat T cells	6
Bid Western Blot in hepatocytes and Jurkat T cells	7
P-JNK Western Blot in hepatocytes and Jurkat T cells	8
Cytotoxicity of TNF-α, FasL and IL-1β in hepatocytes	9
Cytotoxicity of TNF-α, FasL, UV irradiation and IL-1β in Jurkat T cells	10

NF-κB-DNA binding in response to FasL treatment in Jurkat T cells

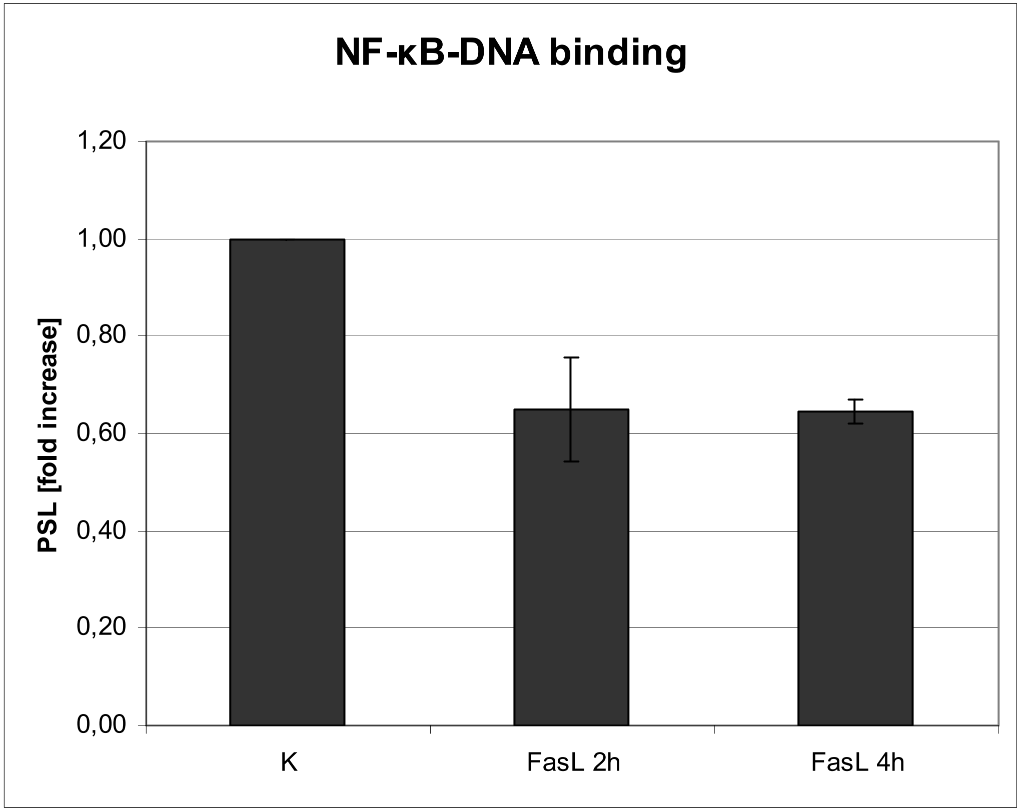

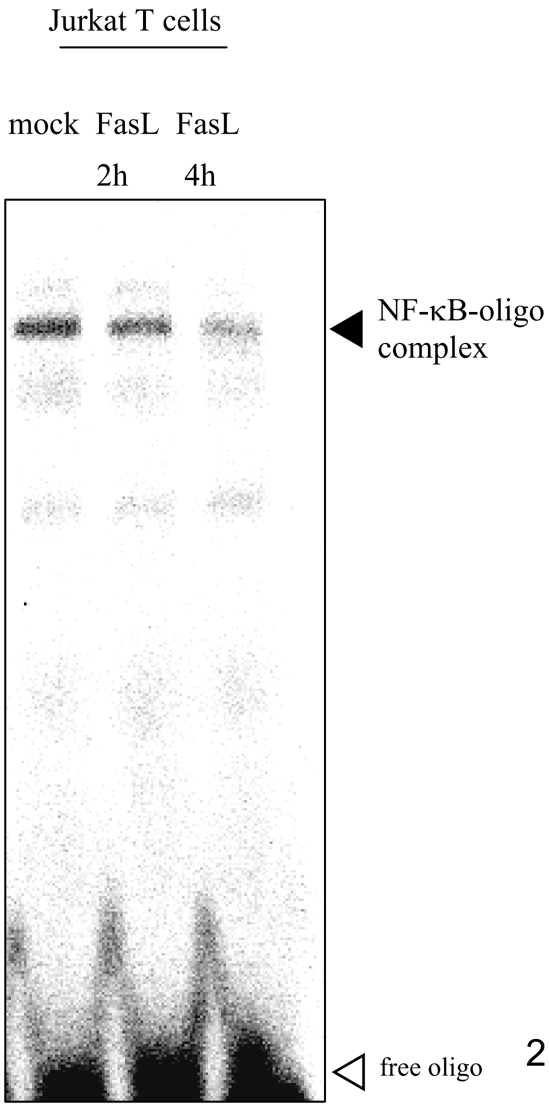

Jurkat T cells were treated with 25ng/ml FasL for the indicated times and equal amounts of nuclear protein were subjected to EMSA. NF-κB-DNA binding is measured as PSL and expressed as fold increase of untreated cells. Means of two independent experiments with sd are shown.

IκB-α Western Blot in hepatocytes and Jurkat T cells

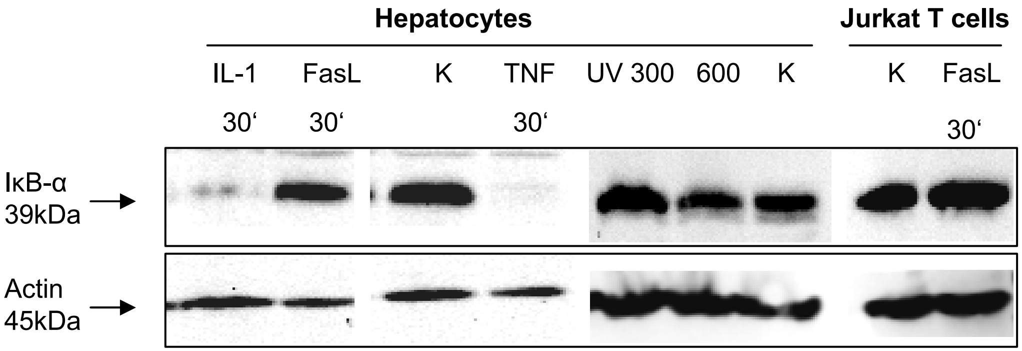

Hepatocytes were stimulated with TNF-α 25ng/ml, IL-1β 20ng/ml, FasL 50ng/ml, UV irradiation 300J/m^2 or 600 J/m^2 and Jurkat Tcells with FasL 50ng/ml, respectively and total extracts were subjected to western blotting targeting IκB-α and after stripping actin as a loading control. Note that cells treated with UV radiation 600 J/m^2 reveal higher IκB-α levels then the model predicted. After UV radiation cells had to be cultivated further before preparing total extracts so that initial NF-κB activity already induced protein newsynthesis of its negative feedback regulator IκB-α. NF-κB is still active in this state which is shown by EMSA in Fig.2.

cFLIP and cIAP2 mRNA levels in Jurkat T cells

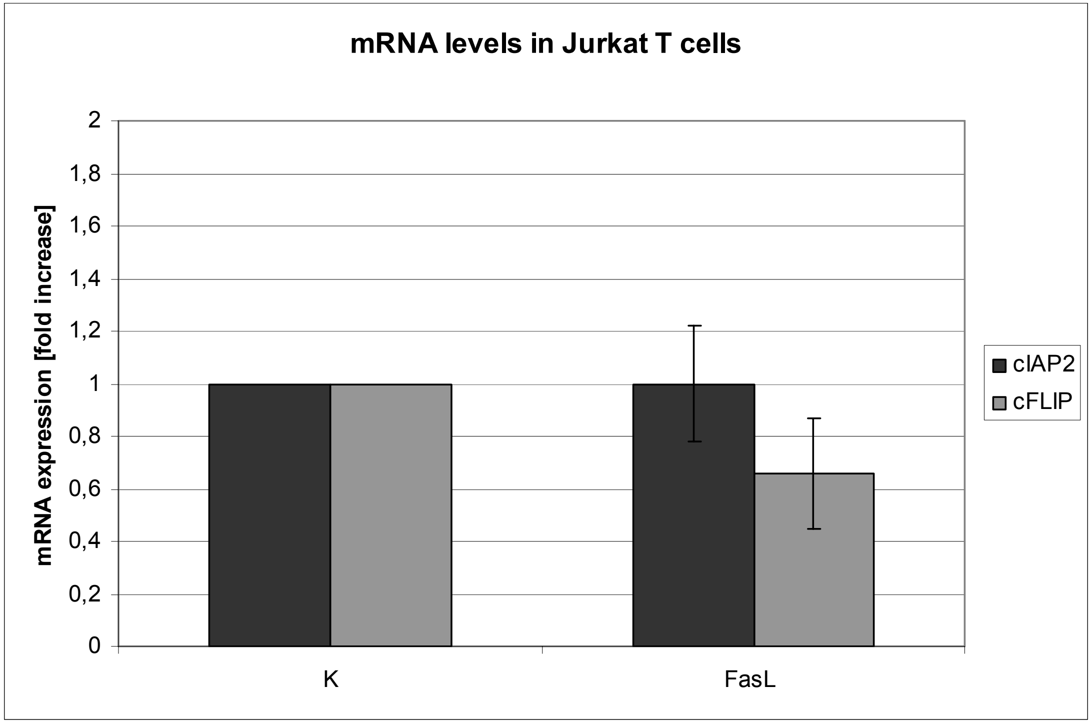

Jurkat T cells were treated with FasL 50ng/ml for 2h, total RNA was isolated and cIAP2 and cFLIP mRNA levels were determined by qRT-PCR. Means of two independent experiments with sd are shown.

XIAP Western Blot in hepatocytes and Jurkat T cells

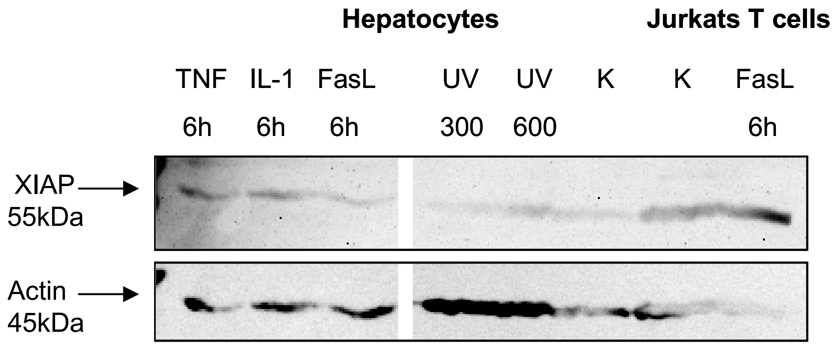

Hepatocytes were stimulated with TNF-α 25ng/ml, IL-1β 20ng/ml, FasL 50ng/ml, UV irradiation 300J/m² or 600 J/m² and Jurkat T cells with FasL 50ng/ml, respectively, for the indicated times. Total extracts were subjected to western blotting targeting XIAP and after stripping actin as a loading control.

Caspase-3 activity in response to FasL and UV irradiation in Jurkat T cells

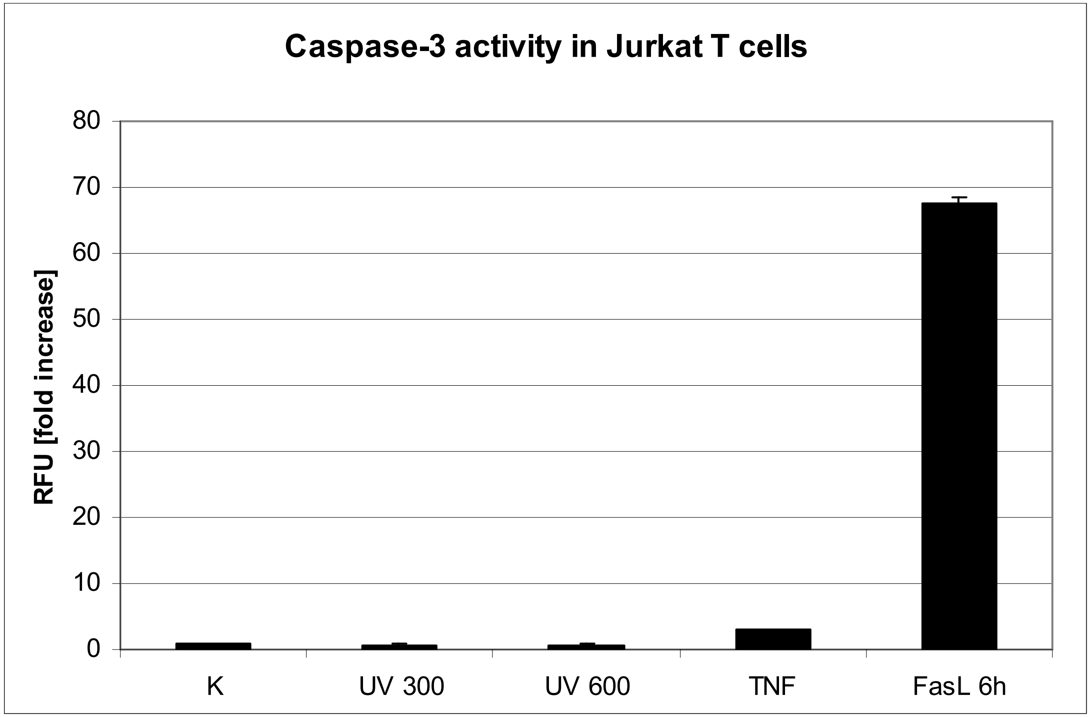

Jurkats T cells were treated with 25ng/ml TNF-α, 50ng/ml FasL, UV irradiation 300 J/m² or 600 J/m² or 100nM insulin for the indicated times and caspase-3 activity was measured.

Bid Western Blot in hepatocytes and Jurkat T cells

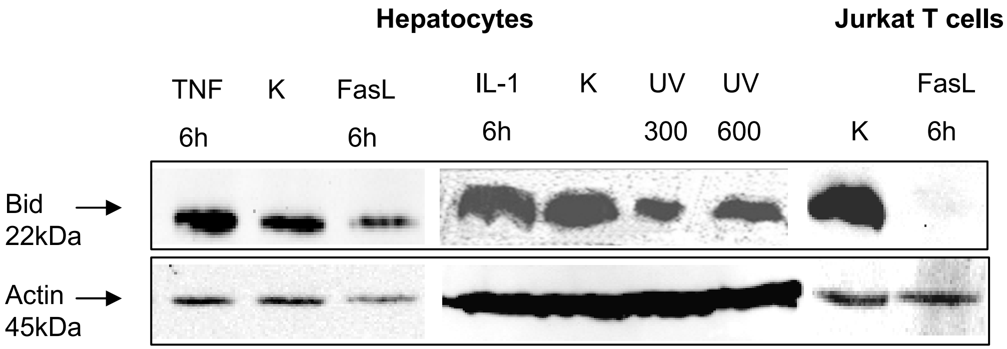

Hepatocytes were stimulated with TNF-α 25ng/ml, IL-1β 20ng/ml, FasL 50ng/ml, UV irradiation 300J/m² or 600 J/m² and Jurkat T cells with FasL 50ng/ml, respectively, for the indicated times. Total extracts were subjected to western blotting targeting Bid and after stripping actin as a loading control.

P-JNK Western Blot in hepatocytes and Jurkat T cells

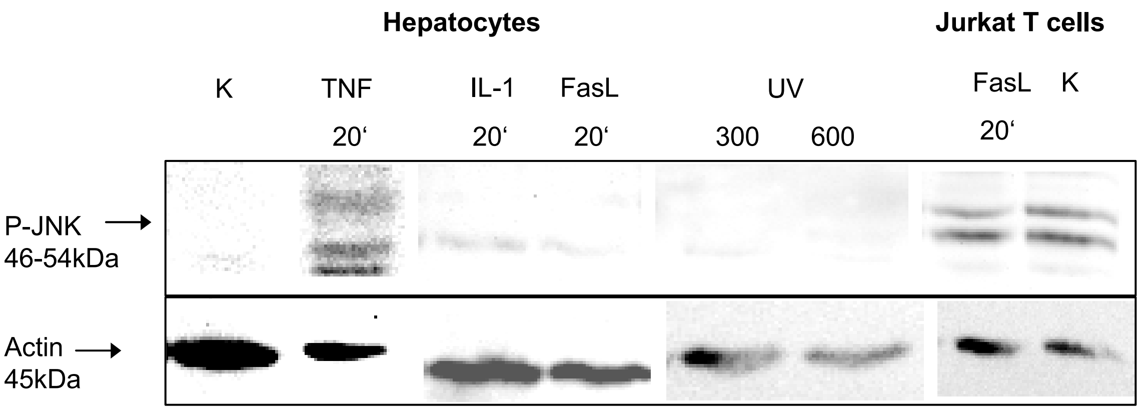

Hepatocytes were stimulated with TNF-α 25ng/ml, IL-1β 20ng/ml, FasL 50ng/ml, UV irradiation 300J/m² or 600 J/m² and Jurkat T cells with FasL 50ng/ml respectively for the indicated times. Total extracts were subjected to western blotting targeting P-JNK and after stripping actin as a loading control.

Cytotoxicity of TNF-α, FasL and IL-1β in hepatocytes

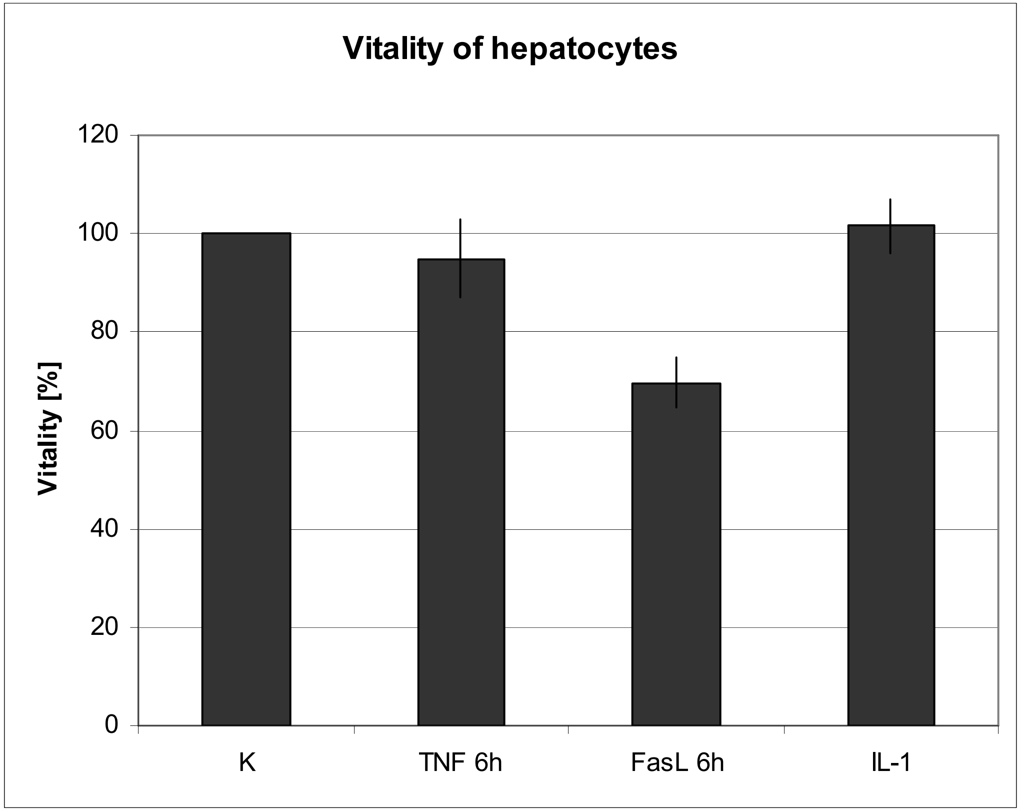

Primary mouse hepatocytes were treated with 25ng/ml TNF-α, 50ng/ml FasL or 20ng/ml IL-1β for the indicated times and vitality was measured by MTT assay and referred to untreated cells. Means of at least 3 independent experiments are shown.

Cytotoxicity of TNF-α, FasL, UV irradiation and IL-1β in Jurkat T cells

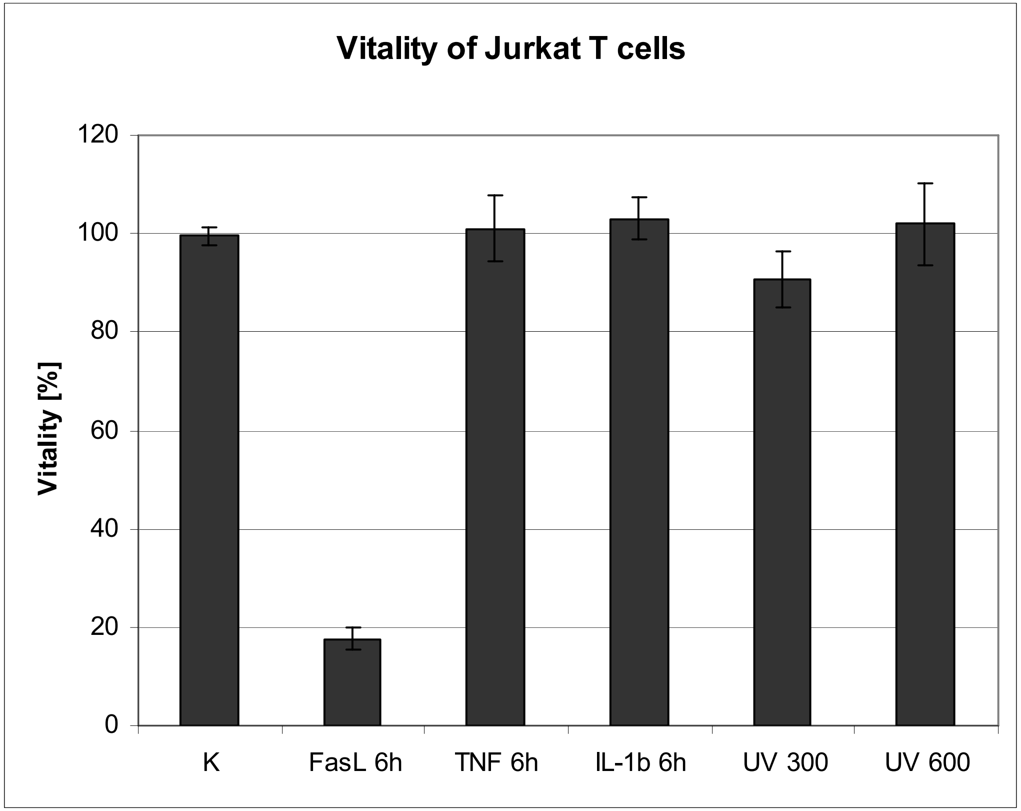

Jurkats T cells were treated with 25ng/ml TNF-α, 50ng/ml FasL, UV irradiation 300 J/m² or 600 J/m² or 20ng/ml IL-1β for the indicated times and vitality was measured by MTT assay and referred to untreated cells. Means of at least 3 independent experiments are shown.
