# Supplementary material for: ON/OFF and Beyond - A Boolean Model of Apoptosis
Source: PLoS Comput Biol. 2009 Dec 11;5(12):e1000595. doi: 10.1371/journal.pcbi.1000595 (PMC2781112; doi:10.1371/journal.pcbi.1000595)
Supplement: Protocol S2 — The file contains a stepwise manual including screenshots how to install CNA and get started with the model. This very short introduction is added for your convenience. Please note that the CNA download contains a comprehensive manual including much more and detailed information which cannot be replaced by this file. (0.58 MB PDF) [file pcbi.1000595.s006.pdf]

How to install CNA and get started – a stepwise introduction

1. Precondition: You need MATLAB® installed on your computer. The MATLAB® license is with cost. However, this is a very common program. If you don't know ask your technical staff whether your institution provides licenses.

2. Install CNA: Go to http://www.mpi-magdeburg.mpg.de/projects/cna/cna.html and scroll down to 'Availability and Download'. CNA is free for academic use. Click the link appropriate for you and follow the instructions. You will receive a folder. Store the folder to any place you like, for example c:\programs\CNA.

3. Store the model on your computer: Unzip the folder 'ApoptosisModel.zip' from the supplementary of this manuscript. Store the folder 'ApoptosisModel' to any place you like but note that the path is not allowed to contain space characters. For example use c:\programs\CNA\ApoptosisModel

4. Start CNA: First start MATLAB®. Change the 'Current Directors' to the path you stored the CNA folder. Afterwards type 'startcna' into the 'Command Window'. Now CNA starts. This was everything you need to do in the MATLAB® user interface. However, CNA displays text regarding some analyses and error messages in the 'Command Window' so you will sometimes come back there to read text.

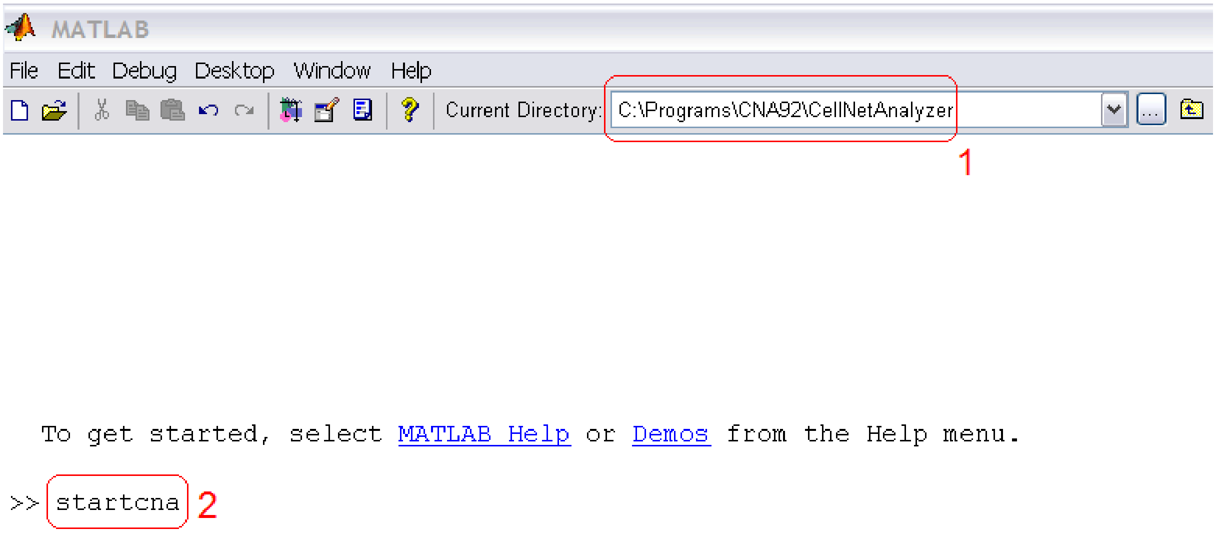

5. Install the apoptosis model:

   a. When CNA starts a new window appears on the screen containing a list of projects which are included in the CNA download to give some examples. Just click 'Project' and choose 'New…'.

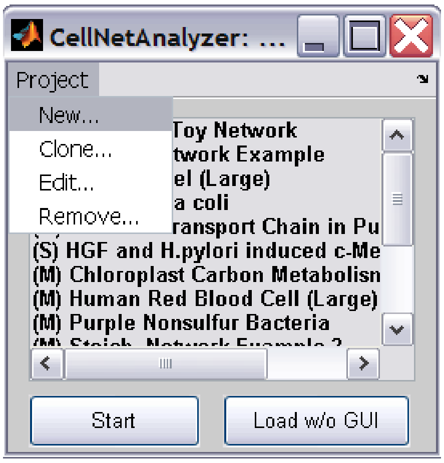

b. The window ‘Declaration of a new network’ appears. Choose a network title, for example ‘Apoptosis’. Type in the path to the model folder, for example c:\programs\CNA\ApoptosisModel. Choose network type ‘Signal-flow’. Choose the file for the network map. You can use the ‘Browse…’ Button therefore. The map is included in the folder ‘ApoptosisModel’ and is named ‘apoptosismap.bmp’. Finally change the adjustment for the text boxes to width 0.01, height 0.02 and fontsize 8 for reactions and compounds. Click the button ‘Save’.

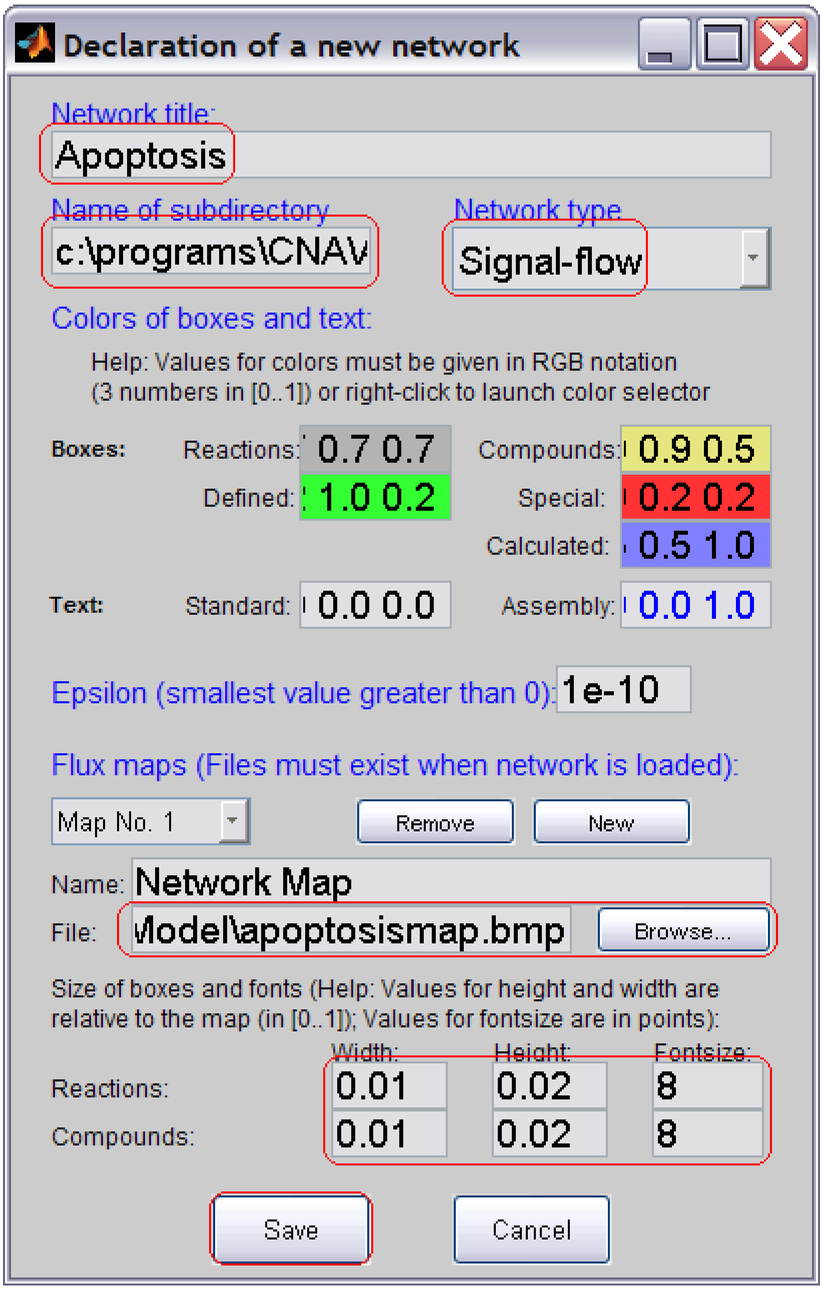

If you did any mistake you can come back to this window by highlighting the network title in the list of projects and choose ‘Edit…’.

6. Start the model: Choose the network title from the list of projects and click the 'Start' button beneath the list. The map opens now and should look like this:

   The red boxes represent the model inputs. The beige one represent nodes and the grey one represent reactions. The only button you necessarily need in the following is 'CellNetAnalyzer' in the upper line framed in red.

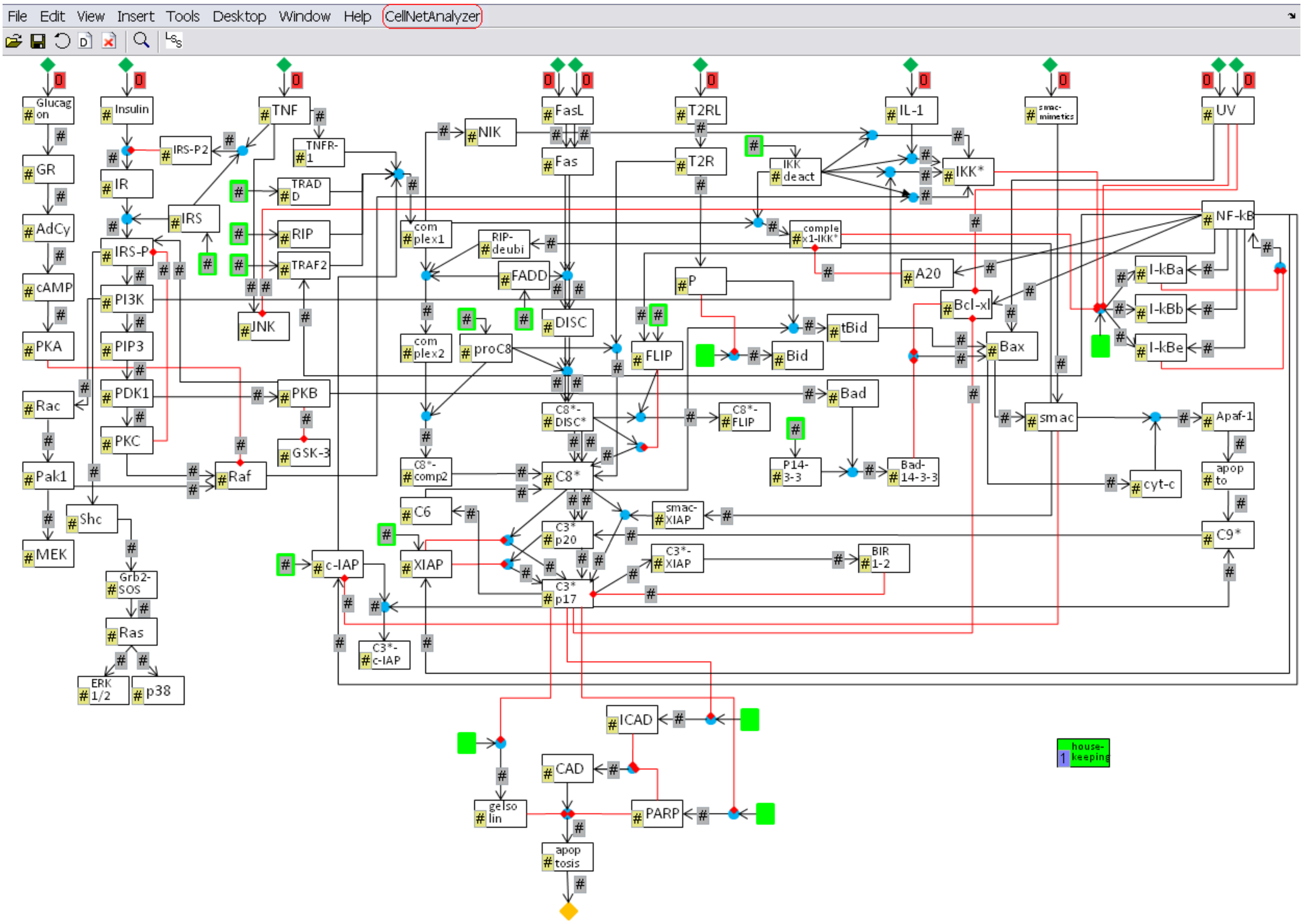

7. You can now use all tools provided by CNA. We recommend reading chapter 4 'Constructing and analyzing of signal-flow networks' of the CNA manual which is included in the CNA folder and also available here: http://www.mpi-magdeburg.mpg.de/projects/cna/manual/toc_frame.htm.

Now we will give an example how to compute a steady state of the model. This is not a necessary step.

1. We strongly recommend to use always the option ‘Set default scenario’ before computing a steady state to be sure that no unintentional values are still in the map.

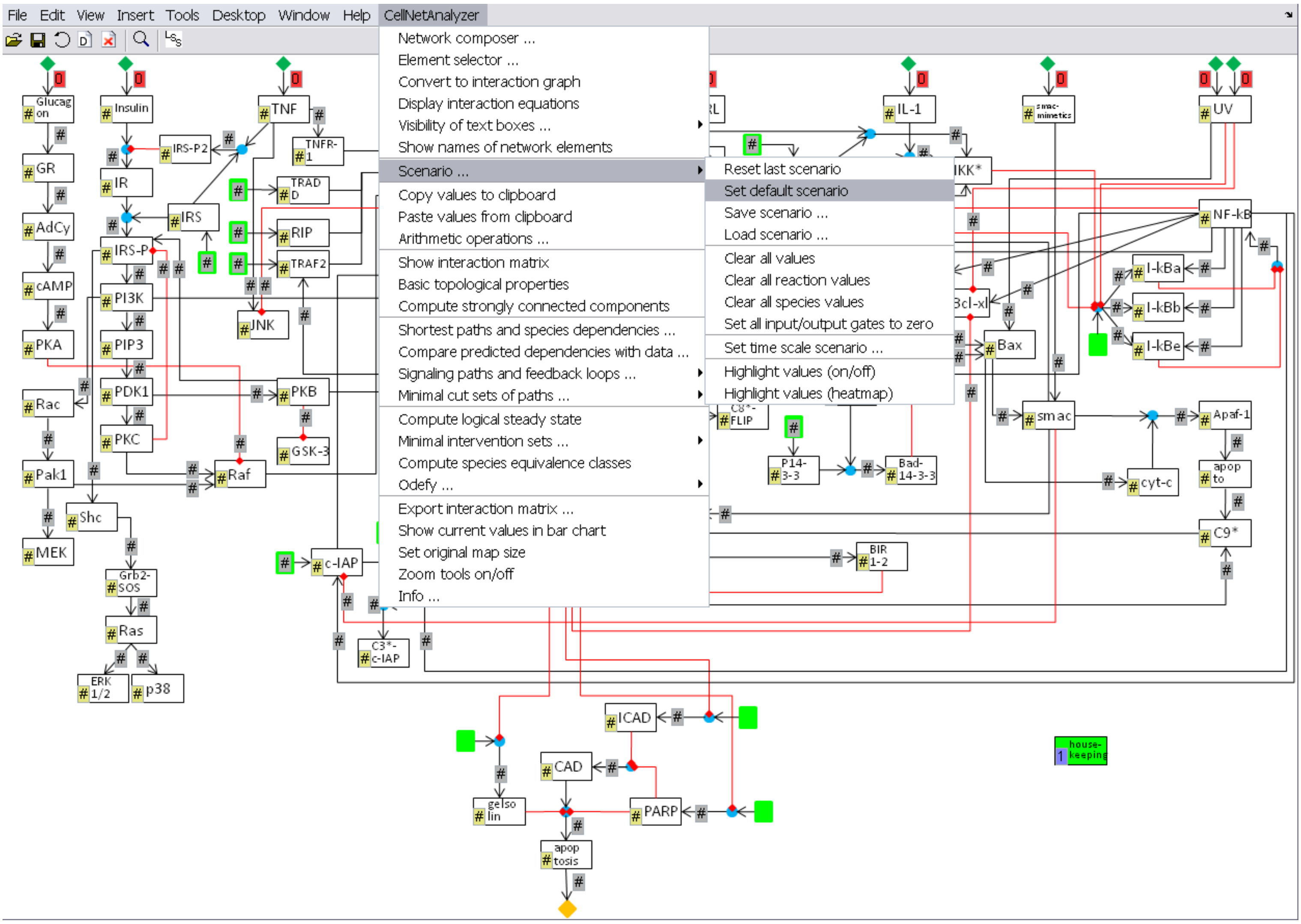

2. Now you can type some values in the map you like. For example we will compute the steady state for a TNF stimulus. Just click in the according box and type in the number ‘1’. Then choose the option ‘Compute logical steady state’ from the ‘CellNetAnalyzer’ drop down bar.

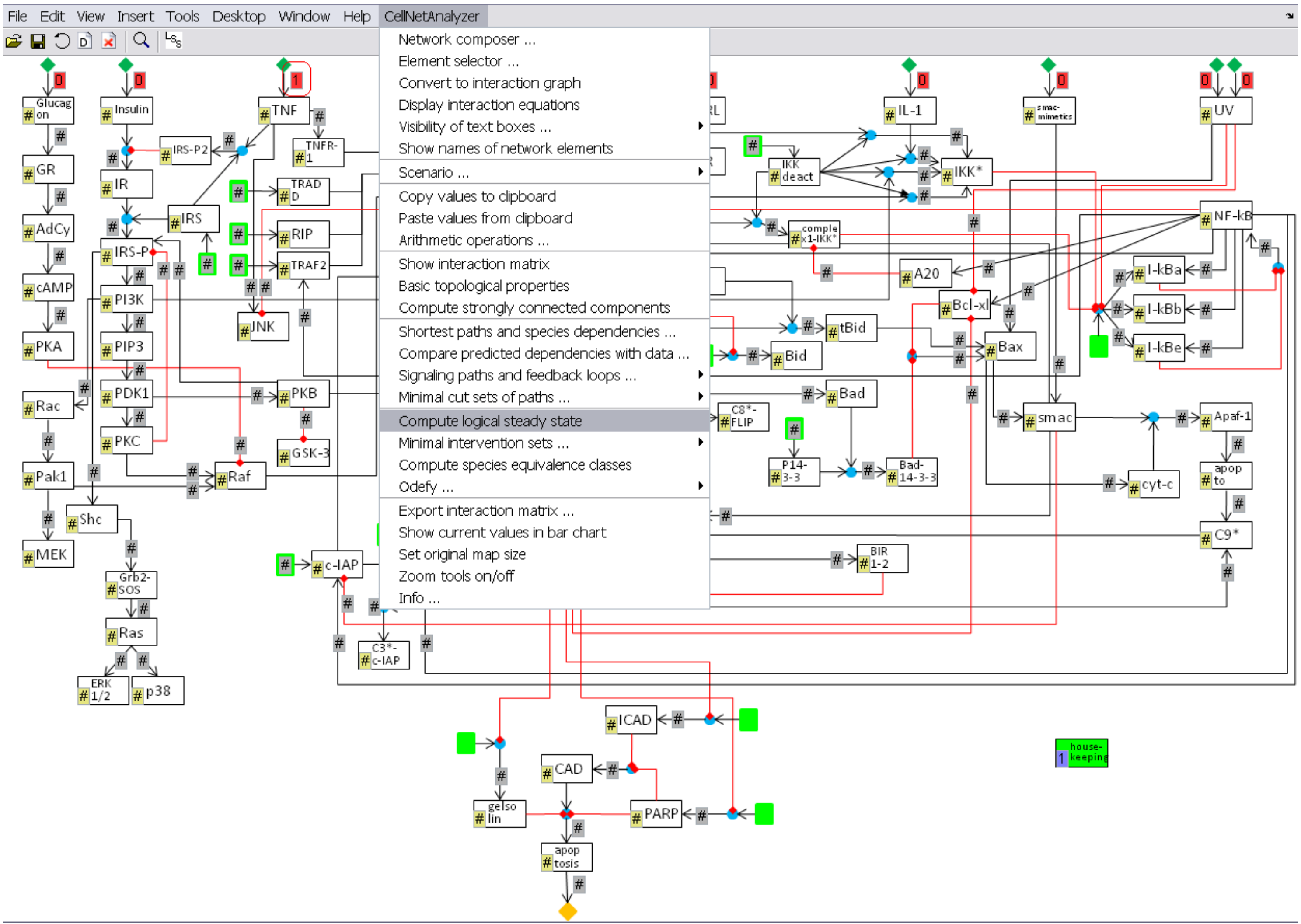

3. CNA computes the logical steady state and the result should look like this:

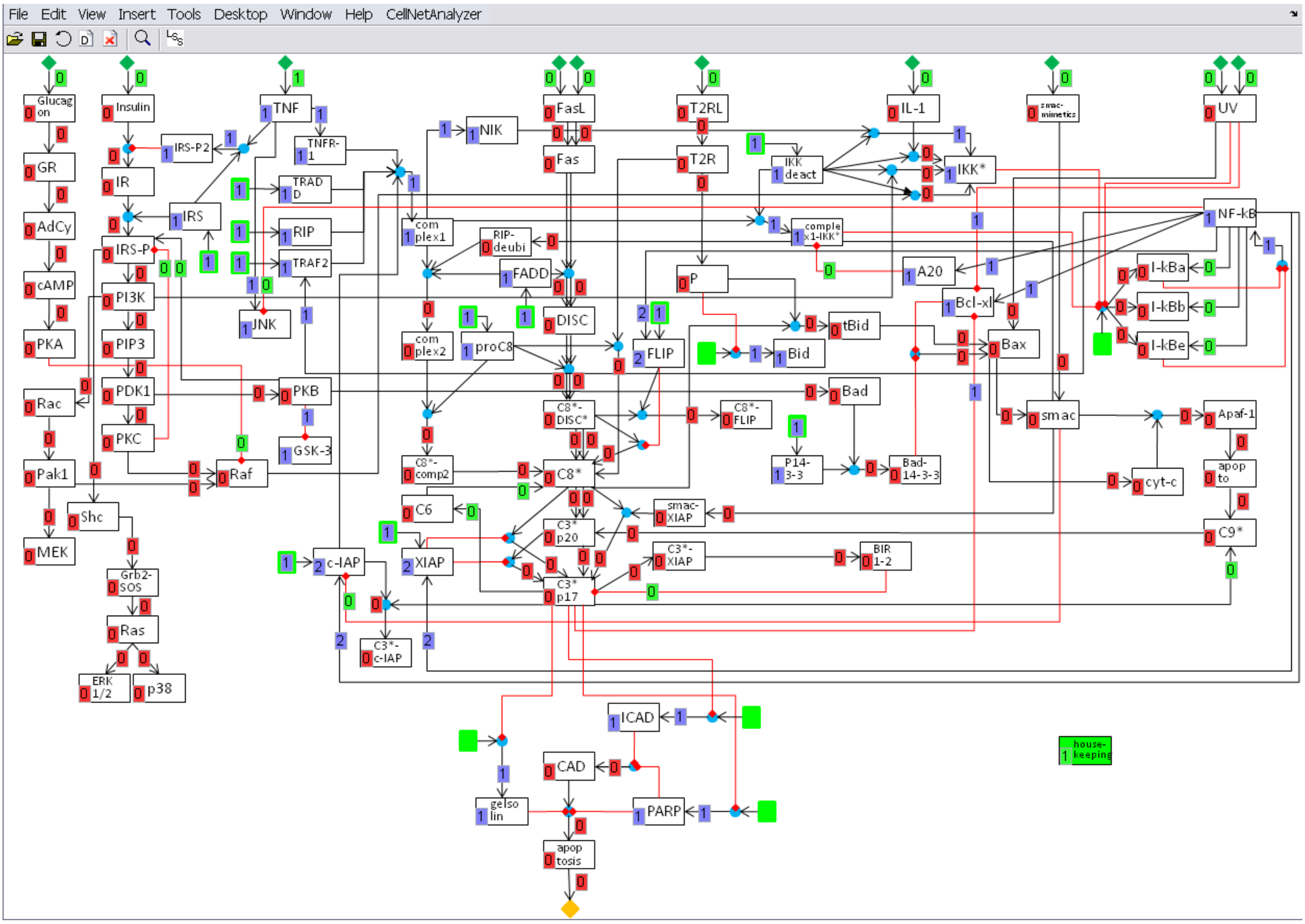

4. Repeat these steps for some other scenarios you are interested in. You can also type numbers in the map for nodes inside the network. Test to type a '0' in a box to simulate a knock-out cell which doesn't contain this protein.

Some finale hints:

- Don't change the size of the network map window, it will look grainy. If the network map looks grainy from the beginning you have to adapt your monitor resolution. An according message will be in your MATLAB® 'Command Window'.
- You can mouse over every box to get additional information. Especially the underlying reactions to the arcs will show up.
- The option 'Network composer…' is the only place you can change the model itself and also 'destroy' it. Apart from this you can just click all over the place as all other actions are not saved in any way.
- The option 'Set time scale scenario…' is located under 'Scenario…' in the 'CellNetAnalyzer' drop-down menu.
- If you get in a mess just close the network map and open it again.
- If you don't see any reaction or miss some output check the MATLAB® 'Command Window'.
- Don't forget that the interaction graph representation is required for computation of signaling pathways, feedback loops and species dependencies because it unambiguously indicates which nodes are involved in interactions. For logical steady state analysis and minimal intervention sets the LIH representation is required to capture all constraints and influences included in the model (compare *Materials and methods* of the manuscript). The transformation is not saved and will be reversed when you open the model the next time.
- If you need further advice to the apoptosis model, want to report bugs or give any kind of feedback please feel free to contact Rebekka Schlatter:

  rebekka.schlatter@isys.uni-stuttgart.de
